# A Pangenome Approach for Discerning Species-Unique Gene Markers for Identifications of *Streptococcus pneumoniae* and *Streptococcus pseudopneumoniae*

**DOI:** 10.3389/fcimb.2020.00222

**Published:** 2020-05-19

**Authors:** Lucia Gonzales-Siles, Roger Karlsson, Patrik Schmidt, Francisco Salvà-Serra, Daniel Jaén-Luchoro, Susann Skovbjerg, Edward R. B. Moore, Margarita Gomila

**Affiliations:** ^1^Department of Infectious Diseases, Institute of Biomedicine, Sahlgrenska Academy, University of Gothenburg, Gothenburg, Sweden; ^2^Department of Clinical Microbiology, Sahlgrenska University Hospital, Gothenburg, Sweden; ^3^Centre for Antibiotic Resistance Research (CARe), University of Gothenburg, Gothenburg, Sweden; ^4^Culture Collection University of Gothenburg (CCUG), Sahlgrenska Academy, University of Gothenburg, Gothenburg, Sweden; ^5^Department of Biology-Microbiology, Universitat de les Illes Balears, Palma, Spain

**Keywords:** *Streptococcus*, pneumococcus, *S. pseudopneumoniae*, pangenome, proteotyping, gene markers, identification

## Abstract

Correct identifications of isolates and strains of the Mitis-Group of the genus *Streptococcus* are particularly difficult, due to high genetic similarity, resulting from horizontal gene transfer and homologous recombination, and unreliable phenotypic and genotypic biomarkers for differentiating the species. *Streptococcus pneumoniae* and *Streptococcus pseudopneumoniae* are the most closely related species of the clade. In this study, publicly-available genome sequences for *Streptococcus pneumoniae* and *S. pseudopneumoniae* were analyzed, using a pangenomic approach, to find candidates for species-unique gene markers; ten species-unique genes for *S. pneumoniae* and nine for *S. pseudopneumoniae* were identified. These species-unique gene marker candidates were verified by PCR assays for identifying *S. pneumoniae* and *S. pseudopneumoniae* strains isolated from clinical samples. All determined species-level unique gene markers for *S. pneumoniae* were detected in all *S. pneumoniae* clinical isolates, whereas fewer of the unique *S. pseudopneumoniae* gene markers were present in more than 95% of the clinical isolates. In parallel, taxonomic identifications of the clinical isolates were confirmed, using conventional optochin sensitivity testing, targeted PCR-detection for the “Xisco” gene, as well as genomic ANIb similarity analyses for the genome sequences of selected strains. Using mass spectrometry-proteomics, species-specific peptide matches were observed for four of the *S. pneumoniae* gene markers and for three of the *S. pseudopneumoniae* gene markers. Application of multiple species-level unique biomarkers of *S. pneumoniae* and *S. pseudopneumoniae*, is proposed as a protocol for the routine clinical laboratory for improved, reliable differentiation, and identification of these pathogenic and commensal species.

## Introduction

*Streptococcus pneumoniae*, also known as “pneumococcus,” is an important human pathogen, causing severe infections, such as meningitis, pneumonia, and bacteremia and responsible for considerable mortality worldwide (O'Brien et al., 2009). Among the pneumococci, a subset of atypical strains have been recognized that are optochin-resistant, bile insoluble, and lacking capsule, an essential virulence factor for disease (Sá-Leão et al., 2006; Rolo et al., 2013). Arbique et al. (2004) described some of these atypical pneumococci as a new species, *Streptococcus pseudopneumoniae*, which is characterized by being bile insoluble and optochin-resistant, when incubated in 5% CO_2_ atmosphere, but optochin–susceptible when incubated under ambient atmosphere.

*Streptococcus pseudopneumoniae* is a recognized member of the Viridans-Group streptococci (VGS) and is closely related, phylogenetically and taxonomically to *S. pneumoniae* and *S. mitis*, sharing nearly 100% 16S rRNA gene sequence similarity (Kilian et al., 2008). The high genotypic similarities between these species are associated with high rates of horizontal gene transfer and homologous recombination between the pathogenic *S. pneumonia*e and commensal Viridans-Group streptococci (Whatmore et al., 2000; Kilian et al., 2008). Because of the close relationships between these species, definitive identifications in the clinical laboratory can be problematic. Phenotypic biochemical and metabolic testing are often insufficient to distinguish *S. pneumoniae*, including atypical *S. pneumoniae*, from *S. pseudopneumoniae* or other closely related streptococci. Molecular-based techniques, such as Multi-Locus Sequence Analysis (MLSA) or Multi-Locus Sequence Typing (MLST), which use sequence analyses of multiple genes or alleles, enabling high resolution differentiation between species, have been shown to be reliable for differentiating *S. pneumoniae* from its closest related species, *S. pseudopneumoniae* (Bishop et al., 2009; Simões et al., 2010). In contrast, individual house-keeping gene PCR- and sequence-based assays have proven to be less reliable for differentiating the other closely related species of the Mitis-Group streptococci from each other (Gonzales-Siles et al., 2019). Several PCR-based methods, mostly targeting genes for specific pneumococcal virulence factors (*lytA, ply, cpsA, pspA*), specific intergenic DNA sequences, and specific regions of the 16S rRNA gene, have been proposed to be pneumococcal species-specific (El Aila et al., 2010; Varghese et al., 2017). Recently, a unique molecular marker, SPS0002, was described (Croxen et al., 2018). However, different studies have shown that these gene markers can be detected also in closely related species, and therefore, are not always reliable for *S. pneumoniae* species-level identifications (Whatmore et al., 2000; Kilian et al., 2008; Johnston et al., 2010; Simões et al., 2010; Rolo et al., 2013).

The availability of whole genome sequence data for a continuously-expanding number of strains of an extensive range of bacterial species makes it increasingly feasible to take advantage of comparative genomic analysis as a tool to recognize the global complexity of bacterial species and for identifying unique biomarkers for taxonomic, as well as functional characterization (Rouli et al., 2015). Pangenome analysis is a comparative whole-genome sequence-based method that enables construction of a framework for assessing the genomic diversity of entire repertoires of genes, as well as identifying core genomic elements. The pangenome includes the “core-genome,” which contains the genes present in all individuals of a lineage or taxon or other defined group, an “accessory or dispensable genome,” formed by the genes not present in all genomes (Medini et al., 2005; Tettelin et al., 2008; Vernikos et al., 2015). Additionally, the pangenome can be divided into four sub-sets, depending on the frequencies with which the genes appear in the different genomes considered: “core,” as defined above; “soft-core,” defined as genes present in at least 95% of the genomes; “shell,” defined as genes which are moderately common; and “cloud,” which represent the genes that are shared by a small fraction of the genomes studied (Koonin and Wolf, 2008; Kaas et al., 2012).

The aim of this study was to identify unique genomic markers, using pangenome analyses, for reliable differentiation and identification of both *S. pneumoniae* and *S. pseudopneumoniae*. For purposes of comparisons, strains of *S. mitis*, which is the next most-closely related species of the Mitis-Group of the genus *Streptococcus*, were also included in this study, to increase the specificities of the gene markers for *S. pneumoniae* and *S. pseudopneumoniae*. The species-dependent presence of these unique markers was confirmed by PCR of reference strains and clinical isolates and validated, using genomic and proteomic approaches.

## Materials and Methods

### Genome Selection

**Figure 1:**
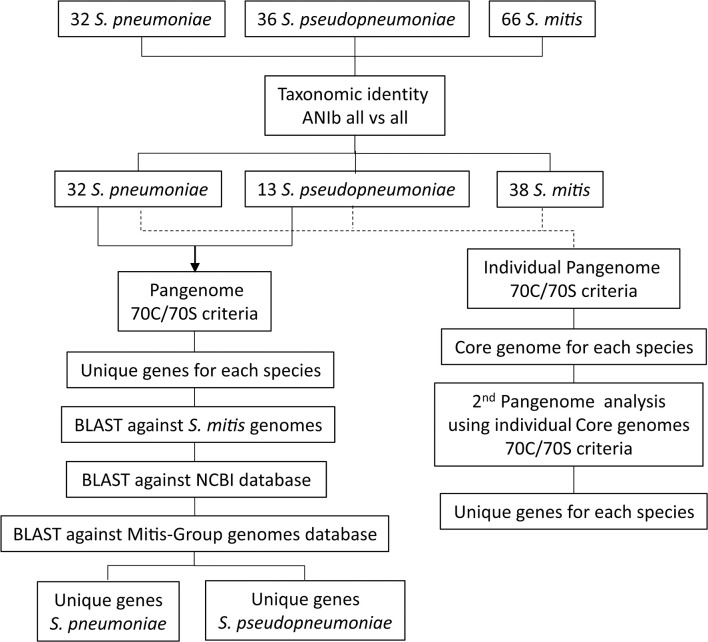
Schematic representation of the workflow used for identifications of unique genes for *S. pneumoniae* and *S. pseudopneumoniae* by applying a pangenome approach.

All complete, closed genome sequences for *Streptococcus pneumoniae* (*n* = 32) and all genome sequences available in GenBank (September 19, 2017) for *S. pseudopneumoniae* (*n* = 36, including one closed genome) and *S. mitis* (*n* = 65, including five closed genomes) were downloaded and included in the analysis for generating the species pangenomes (Supplementary Table 1). The taxonomic affiliations of each of the 134 bacterial genomes were assessed by calculating pair-wise genomic Average Nucleotide Identity (ANI) similarities between all genome sequences, based on the BLAST algorithm (ANIb) (Goris et al., 2007), using the JspeciesWS online service (Richter et al., 2016). A similarity matrix was generated and used to create a dendrogram of genomic relatedness, using the PermutMatrix software (Caraux and Pinloche, 2005), applying hierarchical clustering, the average linkage algorithm (UPGMA) and Pearson's distance correlation (Gomila et al., 2015). The dendrogram was visualized on-line with the Interactive Tree Of Life (iTOL) (Letunic and Bork, 2016). The workflow from genome selection to the identification of unique gene markers is summarized in Figure 1.

### Core- and Pangenome Analyses

After confirmation of genome taxonomic classifications, species core-genomes of *S. pneumoniae* (*n* = 32), *S. pseudopneumoniae* (*n* = 13), and *S. mitis* (*n* = 38) were determined. All genomes correctly classified were annotated, with the software Prokka, version 1.11 (Seemann, 2014). For each genome, the file containing all protein sequences was used for comparison against the rest of the strains by BLASTP (Altschul et al., 1990). Protein sequences were clustered into homologous groups, using the Get_Homologues software (Contreras-Moreira and Vinuesa, 2013). Two proteins were considered homologous if they fulfilled the “70C/70S” (i.e., 70% Contiguously aligned/70% sequence Similarity) criteria, referring to, at least, 70% similarity in, at least, 70% of the sequence (Tettelin et al., 2005). Using the core genomes of each of the different species, second core and pangenome analyses were calculated, applying the 70C/70S criteria, to identify the unique genes for each of the species. A Venn diagram, based on these results, was generated, using the web tool: http://bioinformatics.psb.ugent.be/webtools/Venn/.

Finally, another pangenome analysis, including only the *S. pneumoniae* and *S. pseudopneumoniae* genome sequences (*n* = 45), was performed, using the Get_Homologues software, employing the 70C/70S criteria. Based on this analysis, a list of unique genes for each species, i.e., present in one species and absent in the other one, was generated. In order to confirm the specificities of these genes, a search of the unique genes listed for each species was performed, using BLASTN version 2.6.0+, firstly, against all *S. mitis* genomes and, secondly, against the NCBI database for prokaryotes. Additionally, the genes shown to be unique for *S. pneumoniae* and *S. pseudopneumoniae*, were analyzed again with BLASTN against an internal database including 328 *S. pneumoniae* genome sequences, including the S*. pneumoniae* type strain, and 248 genome sequences assigned to 12 other species of the Mitis-Group of the genus *Streptococcus*, including the type strains of *S. australis, S. cristatus, S. gordonii, S. infantis, S. massiliensis, S. mitis, S. oralis, S. parasanguinis, S. peroris, S. pseudopneumoniae, S. sanguinis*, and *S. sinensis*. The nucleotide sequences of the genes that were confirmed to be unique for a given species were aligned, using Bionumerics 7.5 (Applied Maths, Sint-Martens-Latem, Belgium), to calculate the pair-wise sequence similarities. The nomenclature used to name each unique gene included the species and locus tag provided during annotation with Prokka.

### Confirmation of Gene Markers

For validation of the suggested unique genes as species biomarkers, 29 strains of *S. pseudopneumoniae*, isolated from clinical samples, characterized, and archived at the Culture Collection University of Gothenburg (CCUG), Gothenburg, Sweden, were analyzed by genotypic- and phenotypic-based methods. The strains were screened by PCR for the presence of the unique gene markers, determined from the pangenome analysis. In parallel, the strains were tested by traditional clinical microbiology methods for identification of *S. pseudopneumoniae*, including optochin-sensitivity testing and absence of virulence genes. Whole-genome sequence analyses were performed for 14 of the 29 *S. pseudopneumoniae* strains.

*Streptococcus pneumoniae* unique genes were confirmed, using 20 strains, isolated from clinical samples, archived in the CCUG, for which genome sequences had been determined. The presence or absence of six of the *S. pneumoniae* unique genes was confirmed by PCR.

The presence or absence of the “Xisco” gene, which has been reported to be specific for *S. pneumoniae* strains (Salvà-Serra et al., 2018), was determined, by PCR, for all *S. pneumoniae* and *S. pseudopneumoniae* strains.

BLASTN analysis for each of the unique gene markers for *S. pneumoniae* and *S. pseudopneumoniae* was performed against 29 whole-genome sequences of *S. pseudopneumoniae* and 42 complete genome sequences of *S. pneumoniae* available in GenBank, November 22, 2019, that were not included in the pangenome analysis (Supplementary Table 2). Additionally, the genome sequences of 20 *S. pneumoniae* (Salvà-Serra et al., 2018) and 14 *S. pseudopneumoniae* clinical strains (Supplementary Table 3) were also included in the analysis. Previous confirmation of the taxonomic affiliations of the genomes was performed, as described above.

### DNA Extraction, Whole-Genome Sequencing, and Assembly

Fourteen *S. pseudopneumoniae* strains that were negative for, at least, one of the proposed gene markers or that produced ambiguous results for the optochin-sensitivity testing were selected for whole genome sequence determination and analysis. Genomic DNA (gDNA) was isolated from bacterial strains of pure-culture, fresh biomass, using a Wizard® Genomic DNA Purification Kit (Promega, Madison, WI, USA). The obtained DNA samples were purified, using the DNA Clean & Concentrator^TM^-100 kit (Zymo Research, Irvine, CA, USA). Isolated and purified gDNA was sequenced, using the Illumina MiSeq platform (Eurofins Genomics, Konstanz, Germany). Sequences were trimmed, using Sickle version 1.33 (Joshi and Fass, 2011), with a Phred quality score threshold of Q30. Paired-end reads were assembled, using SPAdes, version 3.11.1 (Bankevich et al., 2012). Taxonomic affiliations were confirmed by ANIb (Richter and Rosselló-Móra, 2009), using JSpeciesWS (Richter et al., 2016), against the type strains *S. pneumoniae* NCTC 7465^T^ and *S. pseudopneumoniae* CCUG 49455^T^. Deposits of sequences were made to GenBank at NCBI under the accession numbers listed in Supplementary Table 3.

### PCR-Amplification

Specific PCR-amplification primers were designed for six unique *S. pseudopneumoniae* and six *S. pneumoniae* gene markers that had a gene size longer than 500 bp (Table 1). Amplification-primers were tested on strains of the respective species. A detail description of the PCR conditions is presented in the Supplementary Information. Strain CCUG 49455^T^, the type strain of *S. pseudopneumoniae*, was used as positive control for *S. pseudopneumoniae* and negative control for *S. pneumoniae*, whereas strain CCUG 28588^T^, the type strain of *S. pneumoniae*, was used as positive control for *S. pneumoniae* and negative control for *S. pseudopneumoniae*. The type strains of the closest phylogenetically-related species in the Mitis-Group of the genus *Streptococcus* (*S. mitis* CCUG 31611^T^ and *S. oralis* CCUG 13229^T^) were also used as negative controls. The presence of the *S. pneumoniae* virulence genes, *cpsA, lytA*, and *ply*, which have been suggested to be present in other species of the Mitis-Group streptococci, were analyzed by PCR in all *S. pseudopneumoniae* strains, according to Nagai et al. (2001), using the primers listed in Table 1.

**Table 1:** List of PCR-primers used for amplification of unique gene markers of *S. pneumonia*e and *S. pseudopneumoniae*.

**Primer name**	**Gene**	**Target**	**Sequence (5^′^->3^′^)**	**References**
Pseudo_901 –F	*ybhF*	*S. pseudopneumoniae*	ATG ACA ACT GCA AAA CTC G	This study
Pseudo_901 –R		*S. pseudopneumoniae*	CCA TTG ATA GCA CAA CTG AC	This study
Pseudo_902 –F	ABC-2 type transporter	*S. pseudopneumoniae*	TGG CTA CCC TCT AGT TAT TG	This study
Pseudo_902 –R		*S. pseudopneumoniae*	CGA CTA CGG AAA TGT TTC TC	This study
Pseudo_231 –F	*gdpE*	*S. pseudopneumoniae*	ATC AGT TCG GAC TGG AGA	This study
Pseudo_231 –R		*S. pseudopneumoniae*	CGA ATT AGG ATT GGG TTA CTC	This study
Pseudo_899 –F	*ypdB*	*S. pseudopneumoniae*	TAG GGC AAG CTG TAT TTA CG	This study
Pseudo_899 –R		*S. pseudopneumoniae*	TGA CAG AGT TTG ATT CGC A	This study
Pseudo_232 –F	*kdpD*	*S. pseudopneumoniae*	ACA GCC CTG TAT ATT GGT AG	This study
Pseudo_232 –R		*S. pseudopneumoniae*	GTG ATG TGG TGA TTT ATC CTG	This study
Pseudo_228–F	Potassium-transporting	*S. pseudopneumoniae*	CTG TTC AAG CCA ATG GTA G	This study
Pseudo_228 –R		*S. pseudopneumoniae*	ACA TCG GCT TCG GGA TTG	This study
Pneumo_1011 –F	*yfmC*	*S. pneumoniae*	GCA AAT TAC GGT GTA AGT GCT GA	This study
Pneumo_1011 –R		*S. pneumoniae*	TAT TGA AAG TGG TGT TGG AGT GCA	This study
Pneumo_1012 –F	*fepD*	*S. pneumoniae*	AGC AGG TTC TAG TCT TGC CAT AA	This study
Pneumo_1012 –R		*S. pneumoniae*	AAG ACC AAC AGC CAT TTC ATC AC	This study
Pneumo_1013 –F	*yfhA*	*S. pneumoniae*	TCC TGA TAT AAT CGG TGT CAC AAG	This study
Pneumo_1013 –R		*S. pneumoniae*	CAG TTA CAA CAC CTA CTG GAT ATC T	This study
Pneumo_1014 –F	*yusV*	*S. pneumoniae*	CAA ATG GTT GTG GGA AAT CAA CAC T	This study
Pneumo_1014 –R		*S. pneumoniae*	CCC AGA AAG TTC TTC AAC TAG GTT A	This study
Pneumo_1961 –F	*gmuR*	*S. pneumoniae*	TTG GAA GGA GCT GCA AGT AAT G	This study
Pneumo_1962 –R		*S. pneumoniae*	AAG CTT TAG ACT TGT TAG TTT CTG AG	This study
Pneumo_127 –F	Putative ABC transporter	*S. pneumoniae*	GAT TTC CCG CTT CCA CTT TCA C	This study
Pneumo_127 –R		*S. pneumoniae*	CGA AAT AGA GTT GCC ACA GAC AT	This study
5202_cpsA_	*cpsA*	*S. pneumoniae*	GCA GTA CAG CAG TTT GTT GGA CTG ACC	Pai et al., 2006
3202_cpsA_R		*S. pneumoniae*	GAA TAT TTT CAT TAT CAG TCC CAG TC	Pai et al., 2006
5203_ply –	*ply*	*S. pneumoniae*	ATT TCT GTA ACA GCT ACC AAC GA	Salo et al., 1995
3203_ply_–R		*S. pneumoniae*	GAA TTC CCT GTC TTT TCA AAG TC	Salo et al., 1995
5322_lytA –F	*lytA*	*S. pneumoniae*	CAA CCG TAC AGA ATG AAG CGG	Nagai et al., 2001
3322_lytA_–R		*S. pneumoniae*	TTAT TCG TGC AAT ACT CGT GCG	Nagai et al., 2001
Spne-CW-F2	“Xisco” gene	*S. pneumoniae*	TGA CGA TTC TAG GAA AAG ATA CAG	Salvà-Serra et al., 2018
Spne-CW-R		*S. pneumoniae*	AGC AGG TGA CTG GTA GGT AAC	Salvà-Serra et al., 2018

### Optochin Testing

Optochin testing was performed, according to Arbique et al. (2004). The description of the method is presented in the Supplementary Information. *S. pneumoniae* CCUG 28588^T^ and S*. pseudopneumoniae* CCUG 49455^T^ were used as controls.

### Proteotyping Analysis Using Bottom-Up Tandem Mass Spectrometry (LC-MS/MS)

Verification of expression of the selected candidate gene markers was performed, by Liquid Chromatography tandem Mass Spectrometry (LC-MS/MS) proteomics. The protein sequences for all unique genes determined for *S. pseudopneumoniae* and *S. pneumoniae* were extracted from the genomes included in the study. Three strains of *S. pneumoniae* (CCUG 28588^T^, CCUG 7206, and CCUG 35180) and three strains of *S. pseudopneumoniae* (CCUG 49455^T^, CCUG 62647, CCUG 63747) had been analyzed by proteotyping (Karlsson et al., 2015), in a previous study (Karlsson et al., 2018), for identifying species-unique peptide markers. A BLASTP search was done, using the protein sequences of the candidate gene marker products against the species-unique peptides found in that previous study.

### Generation of Peptide Inclusion Lists for LC-MS/MS Analysis

Inclusion lists of species-unique peptides were generated for the three most prevalent, by PCR, unique genes of *S. pseudopneumoniae* and for the four most prevalent unique genes of *S. pneumoniae*. The amino acid sequences of the selected genes were digested, *in-silico*, with the enzyme trypsin, using the Peptide Cutter tool at Expasy website (https://web.expasy.org/peptide_cutter/). Peptides ranging from 6 to 25 amino acids in length were selected as these were of suitable length for detection by MS; the MS platform used was the Q Exactive (Thermo Fisher). Inclusion lists were generated, using the MacCoss Lab Software Skyline (https://skyline.ms/project/home/begin.view), and by selecting +2 and +3 as the possible charge states, followed by exporting an isolation list.

The *S. pneumoniae* (CCUG 28588^T^, CCUG 7206, and CCUG 35180) and *S. pseudopneumoniae* (CCUG 49455^T^, CCUG 62647, CCUG 63747) strains were cultivated and analyzed, according to Karlsson et al. (2018) for LC-MS/MS proteotyping. Inclusion lists containing the candidate peptide biomarkers were used during the MS analysis; by employing the inclusion lists, the *m/z* ratios corresponding to these peptide biomarkers were selected for fragmentation even if they were not among the most abundant peptides. Tandem MS data was evaluated, using TCUP (Typing and Characterization Using Proteomics), according to Boulund et al. (2017). In this study, the evaluation was performed at the species level and, thus, sets of species-unique peptides were identified from each strain analysis.

## Results

### Phylogenetic Assignation of Genomes

Taxonomic identifications of the 133 genomes included in the study, by ANIb similarity analyses, confirmed that all 32 *S. pneumoniae* genomes were correctly classified and were, therefore, included in the study. However, only 13 of the 36 S. *pseudopneumoniae* genomes in GenBank were confirmed, by ANIb similarity analyses, as *S. pseudopneumoniae*; 15 of the *S*. “*pseudopneumoniae”* genomes were identified as *S. mitis* and the remaining eight were identified as *S. oralis*. Among the 65 *S. mitis* genomes listed in GenBank, three genomes were identified as *S. oralis*, whereas the remaining 63 were confirmed to be *S. mitis*. After genome sequence-based taxonomic designations of all the selected genomes, 78 genomes were classified as *S. mitis* (Supplementary Table 1). In order to reduce the number of *S. mitis* genomes for the analysis and to attain approximate equality in the number of genomes analyzed for each species, two criteria were implemented. Firstly, genomes were selected, requiring a baseline of 40 or less contigs and, secondly, the selected genomes should cover the representative clades for *S. mitis* depicted in the dendrogram, based on ANIb analysis (Supplementary Figure 1). Based on these criteria, 38 strains of *S. mitis* were included in the analyses (Supplementary Table 1).

### Pangenome Analysis

The pangenome analysis for each of the species showed that *S. pneumoniae* has the highest percentage of genes forming the core genome (35.0%), which are the genes shared by all genomes, compared to *S. pseudopneumoniae* (31.1%) and to *S. mitis* (19.5%). On the other hand, *S. mitis* was observed to have the highest percentage of genes forming the cloud group (55.4%), compared to *S. pneumoniae* (33.0%) and *S. pseudopneumoniae* (43.2%). The fact that *S. mitis* has the lowest relative number of core genes, but more than 50% of cloud genes, suggests that *S. mitis* is, genomically, a relatively heterogeneous species. In Table 2, the pangenome distribution of the genes for each species is indicated.

**Table 2:** Pangenome distribution of the genes for each species, using 70C/70S (70% Contiguously aligned/70% sequence Similarity) criteria.

	***S. mitis***		***S. pneumoniae***		***S. pseudopneumoniae***	
	***n*** **=** **36**		***n*** **=** **32**		***n*** **=** **13**	
	***n***	**%**	***n***	**%**	***n***	**%**
Core	1,007	19.5	1,306	35.0	1,169	31.1
Soft-core	1,196	23.2	1,495	40.1	1,614	42.9
Cloud	2,856	55.4	1,232	33.0	1,626	43.2
Shell	1,101	21.4	1,004	26.9	522	13.9
Pangenome	5,153		3,731		3,762	

**Figure 2:**
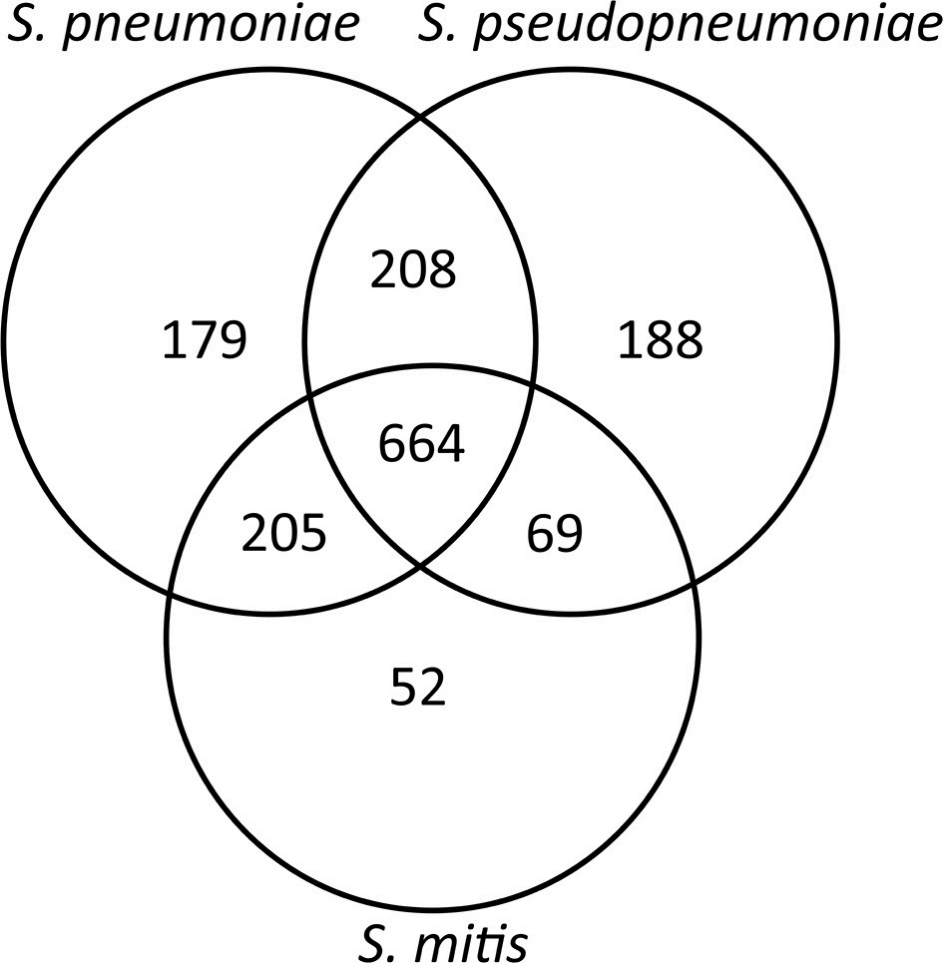
Venn diagram showing the number of shared and unique genes between *S. pneumoniae*. *S. pseudopneumoniae* and *S. mitis*.

The second core genome analysis, including only the genes belonging to species core genomes, obtained by individual analysis for each of the species, showed the number of unique genes for *S. pneumoniae* (*n* = 179) and *S. pseudopneumoniae* (*n* = 188) and a markedly lower number of unique genes for *S. mitis* (*n* = 52; Figure 2). These results are concordant with the high genomic intra-species variation among *S. mitis* genomes shown in previous studies (Kilian et al., 2008, 2014; Jensen et al., 2016).

Due to the low number of unique genes for *S. mitis* showed in the pangenome analysis, a second pangenome analysis was done, including only *S. pneumoniae* and *S. pseudopneumoniae* genome sequences. This analysis showed that 16.7% of the genes were present in the core and 46.4% were present in the cloud (Supplementary Table 4). Although *S. pneumoniae* and *S. pseudopneumoniae* are phylogenetically closely related, a high percentage of genes was observed to be strain-unique and only a small percentage of genes was present in all genomes of a species (Supplementary Table 4).

**Figure 3:**
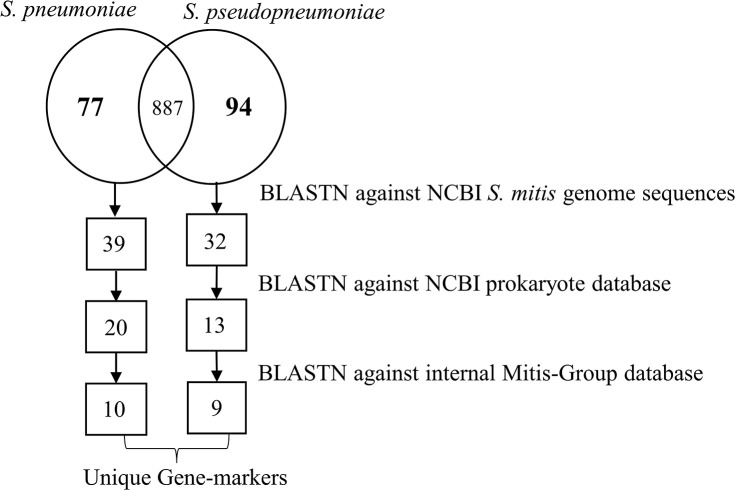
Schematic representation of the number of unique genes found at each step of the analysis for identifications of specific unique biomarkers for *S. pneumoniae* and *S. pseudopneumoniae*.

Based on the pangenome analyses, 94 genes of *S. pseudopneumoniae* and 77 genes of *S. pneumoniae* were observed to be species-unique genes, i.e., genes that are present in all the genomes of one species and absent in all genomes of other species. BLASTN analyses of these proposed unique genes, against the sequences of *S. mitis* genomes, demonstrated that 32 of the 94 genes of *S. pseudopneumoniae* and 39 of the 77 genes of *S. pneumoniae* were not present among any *S. mitis* genomes and were considered potential specific biomarkers for *S. pseudopneumoniae* and for *S. pneumoniae*. A second BLASTN analysis of these candidate genes against the NCBI prokaryote database revealed only 13 of the 32 genes were unique for *S. pseudopneumoniae* and 20 of the 39 genes were unique for *S. pneumoniae* (Figure 3). The discarded genes matched to other *Streptococcus* species and the *Streptococcus* phage IPP62. Furthermore, BLASTN analyses were performed against our internal database. The 13 *S. pseudopneumoniae* unique genes were analyzed, with respect to 567 non-*S. pseudopneumoniae* streptococcal genome sequences, whereas the 20 *S. pneumoniae* unique genes were analyzed with respect to 248 non-*S. pneumoniae* streptococcal genome sequences. From these analyses, nine genes were observed to be unique for *S. pseudopneumoniae* and ten genes unique for *S. pneumoniae* (Table 3) and could be considered potential gene biomarkers for these species. In general, the unique genes for both *S. pseudopneumoniae* and *S. pneumoniae* were observed to be highly conserved, as shown in Table 3.

**Table 3:** List of unique genes of *S. pseudopneumoniae* and *S. pneumoniae*.

	**Gene**	**Protein ID**	**Name**	**Length (bp)**	**Similarity (%)**
*S. pseudopneumoniae*
	Pseudo_232	WP_000847726.1	Potassium-transporting ATPase C chain	2,634	99.8
	Pseudo_901	WP_000205301.1	Putative ABC transporter ATP-binding protein YbhF	912	99.9
	Pseudo_231	WP_000787304.1	KDP operon transcriptional regulatory protein KdpE	727	99.8
	Pseudo_902	WP_000191422.1	ABC-2 type transporter	724	100.0
	Pseudo_899	WP_000912214.1	Transcriptional regulatory protein YpdB	708	99.9
	Pseudo_228	WP_001225808.1	Sensor protein KdpC	619	99.3
	Pseudo_1764	WP_023937803.1	Hypothetical protein	318	99.7
	Pseudo_641	WP_000907298.1	Hypothetical protein	240	99.7
	Pseudo_1933	NA	Hypothetical protein	147	98.2
*S. pneumoniae*
	Pneumo_127	WP_000288029.1	Putative ABC transporter ATP-binding protein	1,242	100.0
	Pneumo_1011	WP_000790743.1	Fe(3+)-citrate-binding protein YfmC precursor	1,026	98.3
	Pneumo_1012	WP_000543061.1	Ferric enterobactin transport system permease protein FepD	1,008	99.8
	Pneumo_1013	WP_001180357.1	Putative siderophore transport system permease protein YfhA	1,008	99.9
	Pneumo_1014	WP_000677520.1	Putative siderophore transport system ATP-binding protein YusV	795	99.8
	Pneumo_1961	WP_000105270.1	HTH-type transcriptional regulator GmuR	729	99.7
	Pneumo_1964	WP_000809626.1	Lichenan-specific phosphotransferase enzyme IIB component	310	99.4
	Pneumo_436	WP_001846568.1	Hypothetical protein	237	99.1
	Pneumo_1362	WP_000500399.1	ApaLI-like restriction endonuclease	192	87.6
	Pneumo_1361	WP_001262530.1	ApaLI-like restriction endonuclease	183	99.9

*The names of the genes, the protein IDs and the proteins encoded by the genes are indicated, as well as the sizes of the genes in nucleotides and the percentages of similarity with the respective genes in the genome of the closest related strains*.

*NA, No annotation available*.

### Confirmation of Gene Markers

PCR-amplification primers targeting the six *S. pneumoniae* unique genes longer than 500 bp (Table 1) were designed and tested on 20 strains isolated from clinical samples and classified as *S. pneumoniae* at the CCUG; all strains tested were positive for all six of the genes. Identifications of the strains were confirmed by gDNA ANIb similarities of each of the genomes, with respect to the genome sequence of *S. pneumoniae* type strain (CCUG 28588^T^). ANIb values were observed to be higher than 98.0% in all cases. In addition, all strains were positive for the presence of the “Xisco” gene (Table 4).

**Table 4:** Genotypic and phenotypic characterization of *S. pneumoniae* strains used for confirmation of gene markers.

**Strain**	**PCR analysis**						**Optochin test**		**WGS analysis**
	**Pneumo**	**Pneumo**	**Pneumo**	**Pneumo**	**Pneumo**	**Pneumo**	**Aerobic**	**CO_**2**_**	**“Xisco” gene**	**ANIb (%)**
	**127**	**1011**	**1012**	**1013**	**1014**	**1961**				
CCUG 28588^T^	+	+	+	+	+	+	S	S	+	99.9
CCUG 1350	+	+	+	+	+	+	S	S	+	98.3
CCUG 6798	+	+	+	+	+	+	S	S	+	98.4
CCUG 7206	+	+	+	+	+	+	S	S	+	98.3
CCUG 11780	+	+	+	+	+	+	S	S	+	98.2
CCUG 32672	+	+	+	+	+	+	S	S	+	98.4
CCUG 33774	+	+	+	+	+	+	S	S	+	98.4
CCUG 35180	+	+	+	+	+	+	S	S	+	98.4
CCUG 35229	+	+	+	+	+	+	S	S	+	98.5
CCUG 35272	+	+	+	+	+	+	S	S	+	98.2
CCUG 35561	+	+	+	+	+	+	S	S	+	98.5
CCUG 36618	+	+	+	+	+	+	S	S	+	98.4
CCUG 36800	+	+	+	+	+	+	S	S	+	98.4
CCUG 45673	+	+	+	+	+	+	S	S	+	98.3
CCUG 63093	+	+	+	+	+	+	S	S	+	98.3
CCUG 63665	+	+	+	+	+	+	S	S	+	98.4
CCUG 68718	+	+	+	+	+	+	S	S	+	98.2
CCUG 69380	+	+	+	+	+	+	S	S	+	98.4
CCUG 69381	+	+	+	+	+	+	S	S	+	98.3
CCUG 69382	+	+	+	+	+	+	S	S	+	98.3

*ANIb values calculated with JSpeciesWS against the type strain of S. pneumoniae NCTC 7465^T^ are also indicated*.

Twenty-nine *S. pseudopneumoniae* CCUG strains isolated from clinical samples were analyzed by PCR for six *S. pneumoniae* unique genes longer than 500 bp (Table 1). All strains of *S. pseudopneumoniae* tested were positive for PCR-amplification of the Pseudo_899 gene, whereas 28 strains (97%) were positive for Pseudo_902 and Pseudo_228 genes, 26 strains (90%) were positive for Pseudo_231, 21 strains (73%) were positive for Pseudo_901, and 17 strains (60 %) were positive for Pseudo_232 genes (Supplementary Figure 2). Sixteen of the strains (57%) were positive for PCR-amplification of all six markers analyzed, whereas five strains (17%) were positive for five and four markers, respectively, two strains (7%) were positive for three markers, and one strain was positive for two markers (3%) (Table 5).

**Table 5:** Genotypic and phenotypic characterization of the 29 *S. pseudopneumoniae* strains used for confirmation of gene markers; ANIb similarity values calculated with JSpeciesWS against *S. pseudopneumoniae* type strain CCUG 49455^T^ are indicated if the whole genome sequence was determined.

**Strain**	**PCR analysis**						**Optochin**		**Additional genes**				**WGS**
	**Pseudo_901**	**Pseudo_902**	**Pseudo_231**	**Pseudo_899**	**Pseudo_232**	**Pseudo_228**	**Aerobic**	**CO_**2**_**	**“Xisco”**	***lytA***	***ply***	***cpsA***	**ANIb (%)**
CCUG 47366	+	+	+	+	+	+	S	R	–	–	+	–	98.6
CCUG 48465	+	+	+	+	+	+	S	R	–	+	+	–	*
CCUG 49455^T^	+	+	+	+	+	+	S	R	–	–	+	–	*
CCUG 50866	+	+	+	+	+	+	S	R	–	–	+	–	*
CCUG 50867	+	+	+	+	+	+	S	R	–	+	+	–	*
CCUG 50868	+	+	+	+	–	+	S	R	–	–	+	–	97.3
CCUG 50869	+	+	+	+	+	+	S	R	–	+	+	–	*
CCUG 50870	+	+	+	+	+	+	S	R	–	–	+	–	*
CCUG 50871	–	+	+	+	–	+	R	R	–	–	+	–	98.8
CCUG 61551	–	+	+	+	–	+	S	R	–	–	+	–	98.2
CCUG 62647	+	+	+	+	+	+	R	R	–	–	+	–	*
CCUG 63747	+	+	+	+	+	+	S	R	–	–	+	–	*
CCUG 63793	–	+	–	+	–	+	S	S	–	–	+	–	97.5
CCUG 64062	–	+	+	+	–	+	S	R	–	–	+	–	96.8
CCUG 69906	+	+	+	+	+	+	S	R	–	+	+	–	*
CCUG 70658	+	+	+	+	+	+	S	R	–	+	+	–	*
CCUG 70988	–	+	+	+	–	+	S	S	–	–	–	–	97.6
CCUG 71653	–	+	–	+	–	+	S	R	–	–	+	–	98.6
CCUG 71770	–	+	+	+	–	+	S	R	–	–	+	–	*
CCUG 71776	+	+	+	+	+	+	S	R	–	–	+	–	*
CCUG 71942	+	+	+	+	+	+	S	R	–	–	+	–	*
CCUG 71983	+	+	+	+	+	+	S	R	–	–	+	–	*
CCUG 71996	–	–	–	+	–	+	S	R	–	–	–	–	98.5
CCUG 72029	+	+	+	+	–	+	S	R	–	–	+	–	97.0
CCUG 72040	+	+	+	+	–	+	S	R	–	–	+	–	96.9
CCUG 72012	+	+	+	+	+	+	S	R	–	–	+	–	*
CCUG 72018	+	+	+	+	+	+	S	R	–	–	+	–	97.3
CCUG 72019	+	+	+	+	+	–	R	R	–	–	+	–	98.5
CCUG 72028	+	+	+	+	–	+	S	R	–	–	+	–	96.8

S, sensitive; R, resistant; *Not whole genome sequenced.

The optochin-sensitivity testing for *S. pseudopneumoniae* strains observed 26/29 strains exhibiting optochin-resistance when cultivated with CO_2_ and optochin-sensitivity when cultivated in aerobic conditions. One strain was resistant and two were sensitive with both cultivation conditions (Table 5).

PCR assays for three of the most prevalent virulence genes found in *S. pneumoniae* strains, namely the genes *cpsA, lytA*, and the *ply* were performed on the *S. pseudopneumoniae* strains. None of the *S. pseudopneumoniae* strains tested carried the *cpsA* gene, whereas 5/29 (17%) samples were positive for *lytA* and 26/29 (93%) samples were positive for the *ply* gene. In only five strains both the *lytA* and *ply* genes were present. In contrast, the presence of the “Xisco” gene, which has been shown to be unique for *S. pneumoniae*, was absent in all *S. pseudopneumoniae* strains.

Fourteen strains of *S. pseudopneumoniae* that were negative for, at least, one of the proposed gene markers or produced ambiguous results for the optochin-sensitivity testing were selected for whole genome sequencing analysis. Genome sequencing yielded paired-end reads of 150 bp, distributed in a total output ranging from 1.7 to 2.7 Gb per strain. Draft genome sequences of total lengths between 2.1 and 2.2 Mb, distributed in a number of scaffolds ranging from 55 to 95, were obtained. N50 values ranged from 48 to 91 kb. The GC content of the genome sequences was between 39.7 and 40.0% (Supplementary Table 3). Genome-based identifications of the strains was done by ANIb, with respect to the genome sequence of *S. pseudopneumoniae* type strain (CCUG 49455^T^). ANIb values were observed to be higher than 96%, confirming that all 14 strains tested were *S. pseudopneumoniae* in all cases (Table 5).

The proposed gene markers were analyzed using BLASTN against additional genome sequences of *S. pneumoniae* and *S. pseudopneumoniae* available in GenBank databases in November 2019, but that had not been included in the pangenome analysis (*S. pneumoniae, n* = 42; *S. pseudopneumoniae, n* = 29), as well as the genome sequences of 20 *S. pneumoniae* and 14 *S. pseudopneumoniae* clinical strains. The analysis revealed that Pneumo_1011, Pneumo_1012, Pneumo_1013, and Pneumo_1014 were present in two *S. pseudopneumoniae* genome sequences, whereas Pneumo_1961 and Pneumo_1964 were present in one *S. pseudopneumoniae* genome sequence (Supplementary Table 5). On the other hand, *S. pseudopneumoniae* gene markers were present in all *S. pseudopneumoniae* genome sequences and absent in all *S. pneumoniae* genome sequences analyzed (Supplementary Table 5).

### Identification of Peptide Biomarker Candidates

Expression of the proposed unique gene markers for *S. pneumoniae* and *S. pseudopneumoniae* was assessed by comparing the protein sequence for each of the unique markers to the list of species-specific peptides, for both species, that were generated by shotgun LC-MS/MS analysis (Karlsson et al., 2018). Among the species unique peptides for *S. pneumoniae*, four peptide matches were observed for the gene marker, Pneumo_1011, and one peptide match was found for each of the gene markers, Pneumo_1014, Pneumo_1361, Pneumo_1961, and Pneumo_1964. For *S. pseudopneumoniae*, two peptide matches were found for the gene marker, Pseudo_228, and one peptide match was found for Pseudo_232 and Pseudo_1933 (Supplementary Table 6). Identification of peptides of the candidate gene markers confirms their expression.

The peptide inclusion lists for *S. pneumoniae* targeted four of the unique gene markers that had a peptide match in the initial analysis. When employing these inclusion lists during LC-MS/MS analysis of three *S. pneumoniae* strains (CCUG 7206, CCUG 35180, and CCUG 28588^T^), 14 peptide matches were found for Pneumo_1011, seven for Pneumo_1014, four for Pneumo_1964 and one match for Pneumo_1961. Six of the matches from Pneumo_1011, three from Pneumo_1014, and one from Pneumo_1964 and Pneumo_1961 were present in all three analyzed strains (Table 6).

**Table 6:** List of the peptides detected from the unique genes of *S. pneumoniae* and *S. pseudopneumoniae* when analyzed using inclusion lists and targeted proteomics.

	**Unique gene**	**Peptide sequence**	**Length (aa)**	**CCUG 49455^**T**^**	**CCUG 62647**	**CCUG 63747**
*S. pseudopneumoniae*
	Pseudo_228	DIISGSQNLAPSNPELK	17	x	x	x
		DIPADLVTTSASGLDPEISPESAK	24		x	x
		ELSLLIEENPTISIR	15			x
		LEEIIDKHTVTK	12	x	x	x
		LIGSALIGQEFSSAAFLHGR	20		x	x
		PSAIQYNTYLSEGDPSGQKR	20		x	x
		VQKELSLLIEENPTISIR	18			x
	Pseudo_899	FLNLGQAVFTFTFGK	15			x
	Pseudo_902	ALPFVPSSNLLR	12	x		
				**CCUG 28588**^**T**^	**CCUG 7206**	**CCUG 35180**
*S. pneumoniae*
	Pneumo_1011	VATIAWGNHDVALALGIVPVGFSK	24	x		x
		ANLFDDLDGLNFEAISNSK	19	x		
		INDADVIITYGDDK	14			x
		VLFTMINAADTSK	13	x	x	x
		EISAEEANK	9			x
		ANYGVSADK	9	x	x	x
		EDYDTLSK	8	x	x	x
		IAPVAAYK	8	x		
		GYSGITK	7	x	x	x
		PWQTLWR	7	x		x
		EGDELIK	7	x	x	x
		TLEALQK	7			x
		ALGMEK	6	x	x	x
		DPLLGK	6			x
	Pneumo_1014	HIAILPQSPIIPESITVADLVSR	23	x	x	x
		ANVEDLANNLVEELSGGQR	19	x		x
		DPISNSPLMIPIGK	14	x		x
		PLEGEVLLDNK	11		x	
		GVLPWTEEK	9	x	x	x
		DDLEIINR	8	x	x	x
		SINSYK	6	x		
	Pneumo_1961	ISEDAHSTIDSR	12	x	x	x
	Pneumo_1964	NIHEADVILIGPQIR	15	x	x	x
		EIAGNIPVDTIDMR	14			x
		VLEQALAWIGEIR	13		x	x
		DYGMMNGAK	9		x	

“*X” indicates presence of a particular peptide of the three strains analyzed for each species*.

*In-silico* peptide inclusion lists were generated for the three most prevalent *S. pseudopneumoniae* gene markers. Species-specific peptide matches for the strains CCUG 62647, CCUG 63747, and the type strain CCUG 49455^T^ were obtained by LC-MS/MS targeted proteomics and matched against the peptide inclusion list. Seven peptide matches were detected for Pseudo_228, from which two were present in the three analyzed strains (Table 6). Only one peptide match was detected for Pseudo_899 and Pseudo_902 and, in both cases, the matches were found in only one strain (Table 6).

## Discussion

Correct identifications and differentiation of the commensal *S. pseudopneumoniae* and *S. mitis* from the bacterial pathogen, *S. pneumoniae*, have been difficult due to conflicting results obtained with different types of analyses combined with the homogeneity and overlapping phenotypic traits of these species, which has led to frequent misidentifications of these species. Whole genome DNA sequence-based methods, such as core genome analysis, and *in silico* genomic calculations, such as ANIb similarities, are not always applicable to all laboratories. Importantly, in the present context, the traditional threshold of ANIb similarity values for species delineations does not apply in certain species of commensal *Streptococcus* species, thus other methodological approaches are needed (Jensen et al., 2016; Gonzales-Siles et al., 2019). In this study, we used the high-resolution capacity of genomics to identify specific molecular markers capable of reliably differentiating and identifying *S. pneumoniae* and *S. pseudopneumoniae*.

As a first criterion, only closed genomes sequences of *S. pneumoniae* were included for the analysis, given the high number of genome sequences available in the databases. Using closed genomes increases the quality of the analysis since the complete sets of genes have been determined for each genome. However, the number of genome sequences available for the commensal *S. pseudopneumoniae* and *S. mitis* species is more limited; therefore, all available genomes sequences, including draft genomes, as well as complete genomes, were included. Since a significant proportion of the genomes in the public databases were misclassified, only the 13 genomes that were confirmed to be *S. pseudopneumoniae* were included, whereas, for *S. mitis*, only genomes with <40 contigs and that represented all taxonomic clusters, based on ANIb analysis for the species, were included. The objective was to have similar numbers of genomes, as many as possible, for each species. Even though the correct taxonomic identifications for many of these genomes have been reported previously (Jensen et al., 2016; Gonzales-Siles et al., 2019), the correct taxonomic identities of these genomes have not been corrected in the public databases, to date. Thus, it was essential to confirm the taxonomic identity of each genome included in the study; this is a fundamental and necessary rule that should be followed in all studies relying upon genome sequence data from the public databases.

Pangenome analysis is used mainly to study the diversity and composition of the complete gene repertoire of a given species (Tettelin et al., 2008). In this study, individual pangenome analyses provided hints of the overall genomic variation within each of the species. *S. pneumoniae* and *S. pseudopneumoniae* possessed a similar percentage distribution of genes in each of the pangenome categories, in contrast to *S. mitis*, wherein the results correlated with the observed high genomic heterogeneity among *S. mitis* strains (Kilian et al., 2014; Jensen et al., 2016). The low percentage of genes belonging to the core (group of genes shared by all strains) and the high number of genes comprising the cloud (genes present in small fractions of strains) indicates high genetic intra-species variation among *S. mitis* strains. This intra-species variation is also corroborated by the low ANIb values of most of the *S. mitis* strains, with respect to the type strain of the species, as shown previously (Jensen et al., 2016; Gonzales-Siles et al., 2019). This feature makes finding species-unique genes difficult, given the probability that the size of the core genome will decrease when higher numbers of strains are analyzed. The low number of species-unique genes for *S. mitis*, compared to the two other species when the core genomes of the three strains were combined, clearly shows this intra-species variation.

The main benefit of using a pangenome approach for determination of unique gene markers is that the pangenome method compares the whole repertoire of genes in the genomes within a given species and between different species. An advantage of this method is that it is not based solely on the analysis of reference strains or previously well-characterized genes; instead, it considers any number of genome sequences, which currently are available in public databases. In addition, the 70C/70S criteria used for the pangenome analysis, in comparison with the traditional 50C/50S criteria used in most studies, increased the reliability and specificity for finding species-unique biomarkers, which is highly important when species that are difficult to differentiate are considered.

The validity of the identification of the 10 unique genes for *S. pneumoniae* found in this study is supported by a recent report based on the analysis of more than 7,500 genomes (Kilian and Tettelin, 2019). However, discrepancies in the results of identifications, using different techniques for isolates and strains of *S. pneumoniae* and *S. pseudopneumoniae*, have been observed when reference strains (from the CCUG) isolated from clinical samples were tested. All of the proposed species-unique markers for *S. pneumoniae* were present in all strains tested, in contrast to the *S. pseudopneumoniae* strains, in which the presence of the proposed species-unique biomarkers was variable between strains. The proposed *S. pneumoniae* biomarkers detected in the pangenome analysis were further analyzed in a larger set of genome sequences of our internal database, including 329 *S. pneumoniae* genome sequences, which increased the specificity of these biomarkers. Furthermore, since only limited numbers of *S. pseudopneumoniae* genomes are available, to date, and all of them were included in the pangenome analysis, the validation analysis was important for confirming the specificities of these markers. The observed variation in the presence of the proposed biomarkers among the different strains highlights the importance of using more than one biomarker for identifications of strains and isolates, particularly considering the high genetic exchange between strains of the Mitis-Group of the genus *Streptococcus*.

Among the species-unique genes found for *S. pseudopneumoniae*, three of the gene markers, Pseudo_228 (Potassium-transporting ATPase C), Pseudo_232 (*kdpD*), and Pseudo_231 (*kdpE*) are associated with the transport of potassium. The two-component system KdpD/KdpE governs K+ homeostasis by controlling synthesis of the high affinity K+ transporter KdpFABC. When sensing low environmental K+ concentrations, the dimeric kinase KdpD autophosphorylates *in trans* and transfers the phosphoryl-group to the response regulator KdpE, which subsequently activates kdpFABC transcription (Mörk-Mörkenstein et al., 2017). K+ is the major monovalent cation in bacteria and important for intracellular osmolarity, pH, cell turgor, enzyme activities, gene expression, and communication between cells (Epstein, 2003; Lee et al., 2010; Humphries et al., 2017). Why these genes are unique and conserved among *S. pseudopneumoniae* strains is not known but they may represent an advantage for the species to survive in the local environment of the human upper respiratory tract.

In contrast, four of the species-unique genes for *S. pneumoniae* gene markers, Pneumo_1011 (*yfmC*), Pneumo_1012 (*fepD*), Pneumo_1013 (*yfhA*), and Pneumo_1014 (*yusV*), are associated with the regulation of iron. Iron regulation is important for *S. pneumoniae*, as iron availability within the host environment can “make or break” a successful infection (Glanville et al., 2018). Compared to other bacterial species, *S. pneumoniae* possesses both high intracellular iron and peroxide levels, which is a perceived recipe for cell suicide (Weiser et al., 2001; Echlin et al., 2016). Thus, it is important for the bacteria to prevent its self-produced, extremely high levels of peroxide from reacting with intracellular iron. The way in which this regulation occurs is not well-known as *S*. *pneumoniae* lacks all typical redox-sensing factors known to alert the cell of such danger (Pericone et al., 2003). *S. pneumoniae* produces millimolar quantities of hydrogen peroxide in oxygen-rich environments as a metabolic by product of pyruvate oxidase, for regulating capsule formation, which is important for colonization of the upper respiratory tract (Echlin et al., 2016). Species-unique genes for both *S*. *pneumoniae* and *S*. *pseudopneumoniae* included ABC transporter proteins, that are vital for cell survival, since they function to counteract any undesirable change occurring in the cell; they could also be involved in the regulation of several physiological processes (Poolman et al., 2004). Finally, one of the genes of *S*. *pneumoniae* and three of *S*. *pseudopneumoniae* are hypothetical proteins. Since these genes are species unique, further studies to elucidate their functions are needed.

In order to determine whether the candidate biomarker genes were expressed or not, the presence of the genes was screened, using mass spectrometry “proteotyping” for discovery of species-unique peptide biomarkers (Karlsson et al., 2018). Proteotyping was able to differentiate the three most closely-related species of the Mitis-Group streptococci. More than 250 species-unique peptides were identified for each of the species *S. pneumoniae, S. pseudopneumoniae*, and *S. mitis*, enabling correct identifications, even when bacterial cells from these species were mixed, i.e., simulating a clinical sample (Karlsson et al., 2018).

In this study, the peptides detected by Karlsson et al. (2018) were used to experimentally verify expression of the candidate biomarker genes identified by the pan-genomics concept. *In-silico* digestion of the selected biomarker genes shown to be expressed, by the study of Karlsson et al. (2018), was performed. All possible peptides that, theoretically, could be generated by trypsin digestion and that was of suitable length for detection by the MS, were identified. Subsequently, “inclusion lists” of these peptides were generated containing which ions (mass-to-charge, m/z) to isolate and select for further fragmentation. In normal conditions, the MS normally operates in “Top10” mode, which means that the 10 most-abundant peptides in each scan are selected for fragmentation. By using “inclusion lists,” m/z ratios corresponding to the peptides in the inclusion list will be selected for fragmentation, even if they are not among the Top10 most-abundant peptides. As a result, fragmentation of a low-abundant peptide in the inclusion lists but not among the 10 most-abundant peptides in an MS-scan, is facilitated. The inclusion lists were used in the analysis of three strains each of *S. pneumoniae, S. pseudopneumoniae*, and *S. mitis*, described by Karlsson et al. (2018). The peptides found by this approach, could be considered to be experimentally verified to be expressed and, thus, suitable to be used as species-unique peptides for proteotyping- or other proteomic-based diagnostics of clinical samples, or suitable as the means for alternative detection methodologies.

This study proposes six *S. pneumoniae* unique gene markers (Pneumo_127, Pneumo_1011, Pneumo_1012, Pneumo_1013, Pneumo_1014, and Pneumo_1961), that have been found in all *S pneumoniae* strains analyzed, and three *S. pseudopneumoniae* unique gene markers (Pseudo_228, Pseudo_899, and Pseudo_902), that are present in more than 95% of analyzed *S. pseudopneumoniae* strains and have been experimentally verified to be expressed. However, additional BLASTN analyses performed against 29 additional genome sequences of *S. pseudopneumoniae* have revealed that four of the six gene markers of *S. pneumoniae* are present in two of these genome sequences (strains EL2652N1 and Spain939) and one present in another genome sequence (strain Spain3473). Therefore, identification of *S. pneumoniae* and *S. pseudopneumoniae*, including more than one unique marker, is proposed for reliable identification and separation of the two species.

Interestingly, other, previously reported gene markers for *S. pneumoniae* were not necessarily detected by our pangenomic approach. For instance, Tavares et al. (2019) indicated that “*lytA, piaB*, and SP2020 were found in non-pneumococcal strains.” Also, Carvalho et al. (2007) reported that a *psaA*-based real-time PCR-assay was positive in *S. pseudopneumoniae* strains. Salvà-Serra et al. (2018) proposed a “Xisco” gene-based PCR-assay for detection and identification of *S. pneumoniae* isolates, after analyzing hundreds of pneumococcal and non-pneumococcal genome sequences. Despite the robustness of the assay, in that same publication, they already reported the presence of a fragment (63%) of the gene in one genome of *S. pseudopneumoniae* and one genome of *S. mitis*. These previously-described marker genes did not pass the strict filters applied in this study.

Despite introduction of conjugate vaccines, *S. pneumoniae* remains a major cause of morbidity and mortality worldwide. *S. pseudopneumoniae* is less virulent, although, recent reports suggest the bacterium to be a potential pathogen in individuals having underlying conditions. It is, therefore, crucial for the clinical laboratory to correctly diagnose and differentiate *S. pneumoniae* and *S. pseudopneumoniae* in clinical samples. This is not least important in samples containing abundant commensal flora, such as sputum samples, in which high numbers of close-related species are present. Correct identification is also important for evaluation of introduced pneumococcal vaccines, including assessments of pneumococcal carriage in the healthy child population. Since pneumococci often tend to lyse and die, due to the activation of autolysin enzymes, molecular detection methods are sometimes superior to culture-dependent methods, and can also be applied in low-income settings. The same is true for patient samples collected after antibiotics have been given, where bacterial detection by culture is often negative, also in cases with severe infection.

The occurrence of horizontal gene transfer and homologous recombination between *S. pneumonia*e and commensal Mitis-Group streptococci (Whatmore et al., 2000; Kilian et al., 2008) makes it difficult to rely on the use of single biomarkers for identifications of these species. The proposition of using more than one marker is based on the findings that not all of the proposed species unique gene markers for *S. pseudopneumoniae* were present in all the strains tested. This is also supported by previous findings that markers reported to be unique for *S. pneumoniae* are also found in clinical isolates of other species of the Mitis-Group of the genus *Streptococcus* (Tavares et al., 2019). These facts further demonstrate the importance of using multi-locus approaches for identifications and differentiation of *S. pneumoniae* and *S. pseudopneumoniae*.

## Data Availability Statement

The genome sequences of the 14 *S. pseudopneumoniae* clinical strains determined in this study have been deposited in DDBJ/ENA/GenBank under the following accession numbers: CCUG 47366 (RXXW01), CCUG 50868 (RXXV01), CCUG 50871 (RXXU01), CCUG 61551 (RXXT01), CCUG 63793 (RXXS01), CCUG 64062 (RXXR01), CCUG 70988 (RXXQ01), CCUG 71653 (RXXP01), CCUG 71996 (RXXN01), CCUG 72018 (RXXM01), CCUG 72019 (RXXL01), CCUG 72028 (RXXK01), CCUG 72029 (RXXJ01), and CCUG 72040 (RXXI01).

## Author Contributions

LG-S, EM, and MG conceived the study. LG-S, RK, FS-S, SS, and MG designed the experiments. LG-S, PS, FS-S, DJ-L, RK, and MG performed the experiments and analyzed the data. LG-S, RK, EM, and MG drafted the manuscript. SS, FS-S, and DJ-L provided critical inputs. EM and SS acquired the project funding. MG was responsible for the overall direction of the project. All authors read and approved the final manuscript.

## Conflict of Interest

RK is affiliated with a company, Nanoxis Consulting AB. The remaining authors declare that the research was conducted in the absence of any commercial or financial relationships that could be construed as a potential conflict of interest.

## Abbreviations

ANI: average nucleotide identity
ANIb: average nucleotide identity based on BLAST
WGS: whole genome sequencing
LC-MS/MS: Liquid chromatography tandem-mass spectrometry.

## References

[B1] Altschul S. F. Gish W. Miller W. Myers E. W. Lipman D. J. (1990). Basic local alignment search tool. J. Mol. Biol. 215, 403–410. 10.1016/S0022-2836(05)80360-2 2231712

[B2] Arbique J. C. Poyart C. Trieu-Cuot P. Quesne G. Carvalho Mda G. Steigerwalt A. G.. (2004). Accuracy of phenotypic and genotypic testing for identification of *Streptococcus pneumoniae* and description of *Streptococcus pseudopneumoniae* sp. nov. J. Clin. Microbiol. 42, 4686–4696. 10.1128/JCM.42.10.4686-4696.2004 15472328 PMC522306

[B3] Bankevich A. Nurk S. Antipov D. Gurevich A. A. Dvorkin M. Kulikov A. S.. (2012). SPAdes: a new genome assembly algorithm and its applications to single-cell sequencing. J. Comput. Biol. 19, 455–477. 10.1089/cmb.2012.0021 22506599 PMC3342519

[B4] Bishop C. J. Aanensen D. M. Jordan G. E. Kilian M. Hanage W. P. Spratt B. G. (2009). Assigning strains to bacterial species via the internet. BMC Biol. 7:3. 10.1186/1741-7007-7-3 19171050 PMC2636762

[B5] Boulund F. Karlsson R. Gonzales-Siles L. Johnning A. Karami N. Al-Bayati O.. (2017). Typing and characterization of bacteria using bottom-up tandem mass spectrometry proteomics. Mol. Cell. Proteomics 16, 1052–1063. 10.1074/mcp.M116.061721 28420677 PMC5461537

[B6] Caraux G. Pinloche S. (2005). PermutMatrix: a graphical environment to arrange gene expression profiles in optimal linear order. Bioinformatics 21, 1280–1281. 10.1093/bioinformatics/bti141 15546938

[B7] Carvalho Mda G. Tondella M. L. McCaustland K. Weidlich L. McGee L. Mayer L. W. Steigerwalt A. (2007) Evaluation improvement of real-time PCR assays targeting lytA, ply, psaA genes for detection of pneumococcal. DNA. J Clin Microbiol. 10.1128/JCM.02498-06. 17537936 PMC1951257

[B8] Contreras-Moreira B. Vinuesa P. (2013). GET_HOMOLOGUES, a versatile software package for scalable and robust microbial pangenome analysis. Appl. Environ. Microbiol. 79, 7696–7701. 10.1128/AEM.02411-13 24096415 PMC3837814

[B9] Croxen M. A. Lee T. D. Azana R. Hoang L. M. (2018). Use of genomics to design a diagnostic assay to discriminate between *Streptococcus pneumoniae* and *Streptococcus pseudopneumoniae*. Microb. Genom. 4:e000175. 10.1099/mgen.0.000175 29629856 PMC6113875

[B10] Echlin H. Frank M. W. Iverson A. Chang T. C. Johnson M. D. Rock C. O.. (2016). Pyruvate oxidase as a critical link between metabolism and capsule biosynthesis in *Streptococcus pneumoniae*. PLoS Pathog. 12:e1005951. 10.1371/journal.ppat.1005951 27760231 PMC5070856

[B11] El Aila N. A. Emler S. Kaijalainen T. De Baere T. Saerens B. Alkan E.. (2010). The development of a 16S rRNA gene based PCR for the identification of *Streptococcus pneumoniae* and comparison with four other species specific PCR assays. BMC Infect. Dis. 10:104. 10.1186/1471-2334-10-104 20426878 PMC2874796

[B12] Epstein W. (2003). The roles and regulation of potassium in bacteria. Prog. Nucleic Acid Res. Mol. Biol. 75, 293–320. 10.1016/S0079-6603(03)75008-9 14604015

[B13] Glanville D. G. Han L. Maule A. F. Woodacre A. Thanki D. Abdullah I. T.. (2018). RitR is an archetype for a novel family of redox sensors in the streptococci that has evolved from two-component response regulators and is required for pneumococcal colonization. PLoS Pathog. 14:e1007052. 10.1371/journal.ppat.1007052 29750817 PMC5965902

[B14] Gomila M. Peña A. Mulet M. Lalucat J. García-Valdés E. (2015). Phylogenomics and systematics in *Pseudomonas*. Front. Microbiol. 6:214. 10.3389/fmicb.2015.00214 26074881 PMC4447124

[B15] Gonzales-Siles L. Salvà-Serra F. Degerman A. Norden R. Lindh M. Skovbjerg S.. (2019). Identification and capsular serotype sequetyping of *Streptococcus pneumoniae* strains. J. Med. Microbiol. 68, 1173–1188. 10.1099/jmm.0.001022 31268417

[B16] Goris J. Konstantinidis K. T. Klappenbach J. A. Coenye T. Vandamme P. Tiedje J. M. (2007). DNA-DNA hybridization values and their relationship to whole-genome sequence similarities. Int. J. Syst. Evol. Microbiol. 57, 81–91. 10.1099/ijs.0.64483-0 17220447

[B17] Humphries J. Xiong L. Liu J. Prindle A. Yuan F. Arjes H. A.. (2017). Species-independent attraction to biofilms through electrical signaling. Cell 168, 200–209.e12. 10.1016/j.cell.2016.12.014 28086091 PMC5497501

[B18] Jensen A. Scholz C. F. Kilian M. (2016). Re-evaluation of the taxonomy of the mitis group of the genus *Streptococcus* based on whole genome phylogenetic analyses, and proposed reclassification of *Streptococcus dentisani* as *Streptococcus oralis* subsp. dentisani comb. nov., *Streptococcus tigurinus as Streptococcus oralis* subsp. *tigurinus* comb. nov., and *Streptococcus oligofermentans* as a later synonym of *Streptococcus cristatus*. Int. J. Syst. Evol. Microbiol. 66, 4803–4820. 10.1099/ijsem.0.001433 27534397

[B19] Johnston C. Hinds J. Smith A. Van Der Linden M. Van Eldere J. Mitchell T. J. (2010). Detection of large numbers of pneumococcal virulence genes in streptococci of the mitis group. J. Clin. Microbiol. 48, 2762–2769. 10.1128/JCM.01746-09 20519466 PMC2916619

[B20] Joshi N. A. Fass J. N. (2011). Sickle: A Sliding-Window, Adaptive, Quality-Based Trimming Tool for FastQ Files (Version 1.33). Available online at: https://github.com/najoshi/sickle

[B21] Kaas R. S. Friis C. Ussery D. W. Aarestrup F. M. (2012). Estimating variation within the genes and inferring the phylogeny of 186 sequenced diverse *Escherichia coli* genomes. BMC Genomics 13:577. 10.1186/1471-2164-13-577 23114024 PMC3575317

[B22] Karlsson R. Gonzales-Siles L. Boulund F. Svensson-Stadler L. Skovbjerg S. Karlsson A.. (2015). Proteotyping: proteomic characterization, classification and identification of microorganisms – a prospectus. Syst. Appl. Microbiol. 38, 246–257. 10.1016/j.syapm.2015.03.006 25933927

[B23] Karlsson R. Gonzales-Siles L. Gomila M. Busquets A. Salvà-Serra F. Jaén-Luchoro D.. (2018). Proteotyping bacteria: characterization, differentiation and identification of pneumococcus and other species within the mitis group of the genus *Streptococcus* by tandem mass spectrometry proteomics. PLoS ONE 13:e0208804. 10.1371/journal.pone.0208804 30532202 PMC6287849

[B24] Kilian M. Poulsen K. Blomqvist T. Havarstein L. S. Bek-Thomsen M. Tettelin H.. (2008). Evolution of *Streptococcus pneumoniae* and its close commensal relatives. PLoS ONE 3:e2683. 10.1371/journal.pone.0002683 18628950 PMC2444020

[B25] Kilian M. Riley D. R. Jensen A. Bruggemann H. Tettelin H. (2014). Parallel evolution of *Streptococcus pneumoniae* and *Streptococcus mitis* to pathogenic and mutualistic lifestyles. MBio 5:e01490–14. 10.1128/mBio.01490-14 25053789 PMC4120201

[B26] Kilian M. Tettelin H. (2019). Identification of virulence-associated properties by comparative genome analysis of *Streptococcus pneumoniae, S. pseudopneumoniae, S. mitis*, three *S. oralis* subspecies, and *S. infantis*. mBio 10: e01985–19. 10.1128/mBio.02520-19 31481387 PMC6722419

[B27] Koonin E. V. Wolf Y. I. (2008). Genomics of bacteria and archaea: the emerging dynamic view of the prokaryotic world. Nucleic Acids Res. 36, 6688–6719. 10.1093/nar/gkn668 18948295 PMC2588523

[B28] Lee C. R. Cho S. H. Kim H. J. Kim M. Peterkofsky A. Seok Y. J. (2010). Potassium mediates *Escherichia coli* enzyme IIA^Ntr^-dependent regulation of sigma factor selectivity. Mol. Microbiol. 78, 1468–1483. 10.1111/j.1365-2958.2010.07419.x 21143318

[B29] Letunic I. Bork P. (2016). Interactive tree of life (iTOL) v3: an online tool for the display and annotation of phylogenetic and other trees. Nucleic Acids Res. 44, W242–W245. 10.1093/nar/gkw290 27095192 PMC4987883

[B30] Medini D. Donati C. Tettelin H. Masignani V. Rappuoli R. (2005). The microbial pan-genome. Curr. Opin. Genet. Dev. 15, 589–594. 10.1016/j.gde.2005.09.006 16185861

[B31] Mörk-Mörkenstein M. Heermann R. Gopel Y. Jung K. Gorke B. (2017). Non-canonical activation of histidine kinase KdpD by phosphotransferase protein PtsN through interaction with the transmitter domain. Mol. Microbiol. 106, 54–73. 10.1111/mmi.13751 28714556

[B32] Nagai K. Shibasaki Y. Hasegawa K. Davies T. A. Jacobs M. R. Ubukata K.. (2001). Evaluation of PCR primers to screen for *Streptococcus pneumoniae* isolates and beta-lactam resistance, and to detect common macrolide resistance determinants. J. Antimicrob. Chemother. 48, 915–918. 10.1093/jac/48.6.915 11733479

[B33] O'Brien K. L. Wolfson L. J. Watt J. P. Henkle E. Deloria-Knoll M. Mccall N.. (2009). Burden of disease caused by *Streptococcus pneumoniae* in children younger than 5 years: global estimates. Lancet 374, 893–902. 10.1016/S0140-6736(09)61204-6 19748398

[B34] Pai R. Gertz R. E. Beall B. (2006). Sequential multiplex PCR approach for determining capsular serotypes of *Streptococcus pneumoniae* isolates. J. Clin. Microbiol. 44, 124–131. 10.1128/JCM.44.1.124-131.2006 16390959 PMC1351965

[B35] Pericone C. D. Park S. Imlay J. A. Weiser J. N. (2003). Factors contributing to hydrogen peroxide resistance in *Streptococcus pneumoniae* include pyruvate oxidase (SpxB) and avoidance of the toxic effects of the fenton reaction. J. Bacteriol. 185, 6815–6825. 10.1128/JB.185.23.6815-6825.2003 14617646 PMC262707

[B36] Poolman B. Spitzer J. J. Wood J. M. (2004). Bacterial osmosensing: roles of membrane structure and electrostatics in lipid-protein and protein-protein interactions. Biochim. Biophys. Acta 1666, 88–104. 10.1016/j.bbamem.2004.06.013 15519310

[B37] Richter M. Rosselló-Móra R. (2009). Shifting the genomic gold standard for the prokaryotic species definition. Proc. Natl. Acad. Sci. U.S.A. 106, 19126–19131. 10.1073/pnas.0906412106 19855009 PMC2776425

[B38] Richter M. Rosselló-Móra R. Oliver Glöckner F. Peplies J. (2016). JSpeciesWS: a web server for prokaryotic species circumscription based on pairwise genome comparison. Bioinformatics 32, 929–931. 10.1093/bioinformatics/btv681 26576653 PMC5939971

[B39] Rolo D. Simões A. S. Domenech A. Fenoll A. Liñares J. de Lencastre H.. (2013). Disease isolates of *Streptococcus pseudopneumoniae* and non-typeable *S. pneumoniae* presumptively identified as atypical *S. pneumoniae* in Spain. PLoS ONE 8:e57047. 10.1371/journal.pone.0057047 23437306 PMC3578818

[B40] Rouli L. Merhej V. Fournier P. E. Raoult D. (2015). The bacterial pangenome as a new tool for analysing pathogenic bacteria. New Microb. New Infect. 7, 72–85. 10.1016/j.nmni.2015.06.005 26442149 PMC4552756

[B41] Sá-Leão R. Simões A. S. Nunes S. Sousa N. G. Frazão N. De Lencastre H. (2006). Identification, prevalence and population structure of non-typable *Streptococcus pneumoniae* in carriage samples isolated from preschoolers attending day-care centres. Microbiology 152, 367–376. 10.1099/mic.0.28596-0 16436425

[B42] Salo P. Ortqvist A. Leinonen M. (1995). Diagnosis of bacteremic pneumococcal pneumonia by amplification of pneumolysin gene fragment in serum. J. Infect. Dis. 171, 479–482. 10.1093/infdis/171.2.479 7844395

[B43] Salvà-Serra F. Connolly G. Moore E. R. B. Gonzales-Siles L. (2018). Detection of “Xisco” gene for identification of *Streptococcus pneumoniae* isolates. Diagn. Microbiol. Infect. Dis. 90, 248–250. 10.1016/j.diagmicrobio.2017.12.003 29329755

[B44] Seemann T. (2014). Prokka: rapid prokaryotic genome annotation. Bioinformatics 30, 2068–2069. 10.1093/bioinformatics/btu153 24642063

[B45] Simões A. S. Sá-Leão R. Eleveld M. J. Tavares D. A. Carrico J. A. Bootsma H. J.. (2010). Highly penicillin-resistant multidrug-resistant pneumococcus-like strains colonizing children in Oeiras, Portugal: genomic characteristics and implications for surveillance. J. Clin. Microbiol. 48, 238–246. 10.1128/JCM.01313-09 19906899 PMC2812262

[B46] Tavares D. A. Handem S. Carvalho R. J. Paulo A. C. de Lencastre H. Hinds J.. (2019). Identification of *Streptococcus pneumoniae* by a real-time PCR assay targeting SP2020. Sci. Rep. 9:3285. 10.1038/s41598-019-39791-1 30824850 PMC6397248

[B47] Tettelin H. Masignani V. Cieslewicz M. J. Donati C. Medini D. Ward N. L.. (2005). Genome analysis of multiple pathogenic isolates of *Streptococcus agalactiae*: implications for the microbial “pan-genome”. Proc. Natl. Acad. Sci. U.S.A. 102, 13950–13955. 10.1073/pnas.0506758102 16172379 PMC1216834

[B48] Tettelin H. Riley D. Cattuto C. Medini D. (2008). Comparative genomics: the bacterial pan-genome. Curr. Opin. Microbiol. 11, 472–477. 10.1016/j.mib.2008.09.006 19086349

[B49] Varghese R. Jayaraman R. Veeraraghavan B. (2017). Current challenges in the accurate identification of *Streptococcus pneumoniae* and its serogroups/serotypes in the vaccine era. J. Microbiol. Methods 141, 48–54. 10.1016/j.mimet.2017.07.015 28780272

[B50] Vernikos G. Medini D. Riley D. R. Tettelin H. (2015). Ten years of pan-genome analyses. Curr. Opin. Microbiol. 23, 148–154. 10.1016/j.mib.2014.11.016 25483351

[B51] Weiser J. N. Bae D. Epino H. Gordon S. B. Kapoor M. Zenewicz L. A.. (2001). Changes in availability of oxygen accentuate differences in capsular polysaccharide expression by phenotypic variants and clinical isolates of *Streptococcus pneumoniae*. Infect. Immun. 69, 5430–5439. 10.1128/IAI.69.9.5430-5439.2001 11500414 PMC98654

[B52] Whatmore A. M. Efstratiou A. Pickerill A. P. Broughton K. Woodard G. Sturgeon D.. (2000). Genetic relationships between clinical isolates of *Streptococcus pneumoniae, Streptococcus oralis*, and *Streptococcus mitis*: characterization of “Atypical” pneumococci and organisms allied to *S. mitis* harboring *S. pneumoniae* virulence factor-encoding genes. Infect. Immun. 68, 1374–1382. 10.1128/IAI.68.3.1374-1382.2000 10678950 PMC97291

