# Supplementary material for: A Pangenome Approach for Discerning Species-Unique Gene Markers for Identifications of Streptococcus pneumoniae and Streptococcus pseudopneumoniae
Source: Front Cell Infect Microbiol. 2020 May 19;10:222. doi: 10.3389/fcimb.2020.00222 (PMC7248185; doi:10.3389/fcimb.2020.00222)
Supplement: Supplementary file 1 [file Table_1.pdf]

Supplementary Table S1. List of genome sequences included in the pangenome analyses.

Strain	Accesion number	GenBank species designation	Cluster analysis ANIb	Size (Mb)	Level	Pangenome analysis
70585	CP000918	*S. pneumoniae*	*S. pneumoniae*	2.2	Complete	Yes
670-6B	CP002176	*S. pneumoniae*	*S. pneumoniae*	2.2	Complete	Yes
A66	LN847353	*S. pneumoniae*	*S. pneumoniae*	2.0	Complete	Yes
AP200	CP002121	*S. pneumoniae*	*S. pneumoniae*	2.1	Complete	Yes
ATCC 700669	FM211187	*S. pneumoniae*	*S. pneumoniae*	2.2	Complete	Yes
CGSP14	CP001033	*S. pneumoniae*	*S. pneumoniae*	2.2	Complete	Yes
D39	CP000410	*S. pneumoniae*	*S. pneumoniae*	2.0	Complete	Yes
G54	NC_011072	*S. pneumoniae*	*S. pneumoniae*	2.1	Complete	Yes
gamPNI0373	CP001845	*S. pneumoniae*	*S. pneumoniae*	2.1	Complete	Yes
Hungary19A-6	CP000936	*S. pneumoniae*	*S. pneumoniae*	2.2	Complete	Yes
INV104	FQ312030	*S. pneumoniae*	*S. pneumoniae*	2.1	Complete	Yes
INV200	FQ312029	*S. pneumoniae*	*S. pneumoniae*	2.1	Complete	Yes
JJA	CP000919	*S. pneumoniae*	*S. pneumoniae*	2.1	Complete	Yes
NCTC 7465	LN831051	*S. pneumoniae*	*S. pneumoniae*	2.1	Complete	Yes
NT_110_58	CP007593	*S. pneumoniae*	*S. pneumoniae*	2.3	Complete	Yes
OXC141	FQ312027	*S. pneumoniae*	*S. pneumoniae*	2.0	Complete	Yes
P1031	CP000920	*S. pneumoniae*	*S. pneumoniae*	2.1	Complete	Yes
R6	AE007317	*S. pneumoniae*	*S. pneumoniae*	2.0	Complete	Yes
SNP034156	FQ312045	*S. pneumoniae*	*S. pneumoniae*	2.0	Complete	Yes
SNP034183	FQ312043	*S. pneumoniae*	*S. pneumoniae*	2.0	Complete	Yes
SNP994038	FQ312042	*S. pneumoniae*	*S. pneumoniae*	2.0	Complete	Yes
SNP994039	FQ312044	*S. pneumoniae*	*S. pneumoniae*	2.0	Complete	Yes
SP49	CP018136	*S. pneumoniae*	*S. pneumoniae*	2.2	Complete	Yes
SP61	CP018137	*S. pneumoniae*	*S. pneumoniae*	2.1	Complete	Yes
SP64	CP018138	*S. pneumoniae*	*S. pneumoniae*	2.1	Complete	Yes
SPN032672	FQ312039	*S. pneumoniae*	*S. pneumoniae*	2.1	Complete	Yes
SPN033038	FQ312041	*S. pneumoniae*	*S. pneumoniae*	2.1	Complete	Yes
SPNA45	HE983624	*S. pneumoniae*	*S. pneumoniae*	2.1	Complete	Yes
ST556	CP003357	*S. pneumoniae*	*S. pneumoniae*	2.2	Complete	Yes
SWU02	CP018347	*S. pneumoniae*	*S. pneumoniae*	2.1	Complete	Yes
Taiwan19F-14	CP000921	*S. pneumoniae*	*S. pneumoniae*	2.1	Complete	Yes
TCH8431/19A	CP001993	*S. pneumoniae*	*S. pneumoniae*	2.1	Complete	Yes
TIGR4; ATCC BAA-334	AE005672	*S. pneumoniae*	*S. pneumoniae*	2.2	Complete	Yes
1321	AYRP00000000	*S. pseudopneumoniae*	*S. pseudopneumoniae*	2.1	Contig	Yes
5247	AYRQ00000000	*S. pseudopneumoniae*	*S. pseudopneumoniae*	2.1	Contig	Yes
22725	AYRO00000000	*S. pseudopneumoniae*	*S. pseudopneumoniae*	2.2	Contig	Yes
1172_SPSE	JWAJ00000000	*S. pseudopneumoniae*	*S. infantis*	2.0	Contig	No
1213_SPSE	JVYO00000000	*S. pseudopneumoniae*	*S. mitis*	2.0	Contig	No
126_SPSE	JVWP00000000	*S. pseudopneumoniae*	*S. oralis*	2.1	Scaffold	No
1271.rep1_SPSE	JVWC00000000	*S. pseudopneumoniae*	*S. mitis*	2.1	Scaffold	Yes
1271.rep2_SPSE	JVWB00000000	*S. pseudopneumoniae*	*S. mitis*	2.1	Contig	No
1272_SPSE	JVWA00000000	*S. pseudopneumoniae*	*S. mitis*	2.1	Scaffold	No
144_SPSE	JVSM00000000	*S. pseudopneumoniae*	*S. mitis*	1.8	Contig	Yes

163_SPSE	JVRR00000000	*S. pseudopneumoniae*	*S. mitis*	2.1	Scaffold	No
205_SPSE	JVQA00000000	*S. pseudopneumoniae*	*S. mitis*	1.9	Scaffold	No
276-03	LJHJ00000000	*S. pseudopneumoniae*	*S. pseudopneumoniae*	2.2	Contig	Yes
277_SPSE	JVNG00000000	*S. pseudopneumoniae*	*S. mitis*	2.2	Scaffold	No
289_SPSE	JVMU00000000	*S. pseudopneumoniae*	*S. mitis*	2.1	Scaffold	Yes
294_SPSE	JVMO00000000	*S. pseudopneumoniae*	*S. mitis*	2.0	Scaffold	No
315_SPSE	JVLX00000000	*S. pseudopneumoniae*	*S. mitis*	1.9	Scaffold	No
330_SPSE	AFBD00000000	*S. pseudopneumoniae*	*S. mitis*	2.2	Contig	Yes
338-14	LJHI00000000	*S. pseudopneumoniae*	*S. pseudopneumoniae*	2.2	Contig	Yes
342_SPSE	JVKV00000000	*S. pseudopneumoniae*	*S. infantis*	1.9	Contig	No
380_SPSE	JVJI00000000	*S. pseudopneumoniae*	*S. mitis*	2.1	Contig	No
434_SPSE	JVHE00000000	*S. pseudopneumoniae*	*S. infantis*	2.1	Scaffold	No
445_SPSE	JVGV00000000	*S. pseudopneumoniae*	*S. mitis*	2.1	Scaffold	Yes
469_SPSE	JVFV00000000	*S. pseudopneumoniae*	*S. infantis*	2.1	Scaffold	No
61-14	LJHK00000000	*S. pseudopneumoniae*	*S. pseudopneumoniae*	2.3	Contig	Yes
74_SPSE	JUUZ00000000	*S. pseudopneumoniae*	*S. infantis*	1.8	Contig	No
75_SPSE	JUUO00000000	*S. pseudopneumoniae*	*S. mitis*	2.1	Scaffold	No
843_SPSE	JUQX00000000	*S. pseudopneumoniae*	*S. oralis*	2.1	Scaffold	No
888_SPSE	JUPG00000000	*S. pseudopneumoniae*	*S. mitis*	2.1	Contig	No
ATCC BAA-960	AICS00000000	*S. pseudopneumoniae*	*S. pseudopneumoniae*	2.1	Contig	Yes
CCUG 49455	MWSM00000000	*S. pseudopneumoniae*	*S. pseudopneumoniae*	2.2	Scaffold	Yes
CCUG 62647	MUXQ00000000	*S. pseudopneumoniae*	*S. pseudopneumoniae*	2.1	Scaffold	Yes
CCUG 63747	MUXR00000000	*S. pseudopneumoniae*	*S. pseudopneumoniae*	2.2	Scaffold	Yes
G42	AYRN00000000	*S. pseudopneumoniae*	*S. pseudopneumoniae*	2.1	Contig	Yes
IS7493	NC_015875	*S. pseudopneumoniae*	*S. pseudopneumoniae*	2.2	Complete	Yes
SK674	AJKE00000000	*S. pseudopneumoniae*	*S. pseudopneumoniae*	2.1	Contig	Yes
1042_SPSE	JWFA00000000	*S. mitis*	*S. mitis*	1.9	Contig	No
11/5	AQTT00000000	*S. mitis*	*S. mitis*	1.9	Contig	No
1111_SMIT	JWCV00000000	*S. mitis*	*S. mitis*	2.0	Contig	Yes
1217_SPSE	JVYJ00000000	*S. mitis*	*S. mitis*	2.0	Contig	No
13/39	AQTU00000000	*S. mitis*	*S. mitis*	2.1	Contig	No
168_SPSE	JVRN00000000	*S. mitis*	*S. mitis*	1.9	Contig	Yes
17/34	ASZZ00000000	*S. mitis*	*S. mitis*	1.9	Contig	No
18/56	ATAA00000000	*S. mitis*	*S. mitis*	1.9	Contig	No
21/39	AYRR00000000	*S. mitis*	*S. mitis*	2.0	Contig	No
27/7	AYRS00000000	*S. mitis*	*S. mitis*	1.9	Contig	No
29/42	ATAB00000000	*S. mitis*	*S. mitis*	1.9	Contig	No
321A	LBMT00000000	*S. mitis*	*S. mitis*	2.1	Scaffold	Yes
38_SPSE	JVJJ00000000	*S. mitis*	*S. mitis*	1.9	Contig	Yes
850_SMIT	JUQO00000000	*S. mitis*	*S. mitis*	2.1	Contig	No
ATCC 6249	AEEN00000000	*S. mitis*	*S. oralis*	1.9	Scaffold	No
B_009152_10	NCVN00000000	*S. mitis*	*S. mitis*	2.0	Contig	No
B_5756_13	NCVM00000000	*S. mitis*	*S. mitis*	1.9	Scaffold	Yes
B6	NC_013853	*S. mitis*	*S. mitis*	2.1	Complete	Yes
CCUG 31611	MUYN00000000	*S. mitis*	*S. mitis*	1.8	Scaffold	Yes
CCUG 61082	MUXS00000000	*S. mitis*	*S. mitis*	2.0	Scaffold	Yes

CCUG 63687	MUYO00000000	*S. mitis*	*S. mitis*	2.0	Scaffold	Yes
CMW7705B	LRQR00000000	*S. mitis*	*S. mitis*	1.9	Scaffold	No
DD22	CM003839	*S. mitis*	*S. mitis*	2.2	Scaffold	No
DD26	LQOD00000000	*S. mitis*	*S. mitis*	2.1	Scaffold	No
DD28	KQ970261	*S. mitis*	*S. mitis*	2.2	Scaffold	Yes
F0392	AFUO00000000	*S. mitis*	*S. oralis* subsp. *dentisani*	1.9	Contig	No
KCOM 1350	CP012646	*S. mitis*	*S. mitis*	1.9	Complete	Yes
M3-1	LROU00000000	*S. mitis*	*S. mitis*	2.0	Contig	No
M3-4	ACRL00000000	*S. mitis*	*S. mitis*	2.0	Contig	No
NCTC 10712	JYGN00000000	*S. mitis*	*S. mitis*	1.8	Contig	No
NCTC 12261	AEDX00000000	*S. mitis*	*S. mitis*	1.8	Contig	Yes
OD_310347_11	NCVL00000000	*S. mitis*	*S. mitis*	2.0	Scaffold	No
OD_317805_11	NCVK00000000	*S. mitis*	*S. mitis*	2.1	Scaffold	No
OT25	JYGP00000000	*S. mitis*	*S. mitis*	1.9	Contig	Yes
RH_12363_08	NCVJ00000000	*S. mitis*	*S. mitis*	2.0	Scaffold	Yes
RH_17024_08	NCVI00000000	*S. mitis*	*S. mitis*	1.8	Contig	Yes
RH_17439_08	NCVH00000000	*S. mitis*	*S. mitis*	1.9	Scaffold	Yes
RH_43861_09	NCVG00000000	*S. mitis*	*S. mitis*	1.8	Scaffold	Yes
RH_50275_09	NCVF00000000	*S. mitis*	*S. mitis*	2.1	Scaffold	Yes
RH_50738_11	NCVE00000000	*S. mitis*	*S. mitis*	2.0	Contig	Yes
RH_777_07	NCVD00000000	*S. mitis*	*S. mitis*	2.1	Scaffold	No
RH_8961_10	NCVC00000000	*S. mitis*	*S. mitis*	1.9	Scaffold	Yes
SK1073	AFQT00000000	*S. mitis*	*S. mitis*	2.1	Contig	No
SK1080	AFQV00000000	*S. mitis*	*S. mitis*	1.9	Contig	Yes
SK1126	JPFT00000000	*S. mitis*	*S. mitis*	1.9	Contig	Yes
SK137	JPFS00000000	*S. mitis*	*S. mitis*	2.0	Contig	Yes
SK137	JPFS00000000	*S. mitis*	*S. mitis*	2.0	Contig	No
SK145	JYGS00000000	*S. mitis*	*S. mitis*	2.0	Contig	Yes
SK271	JPGW00000000	*S. mitis*	*S. mitis*	1.9	Contig	Yes
SK321	AEDT00000000	*S. mitis*	*S. mitis*	1.9	Contig	Yes
SK564	AEDU00000000	*S. mitis*	*S. mitis*	2.0	Contig	Yes
SK569	AFUF00000000	*S. mitis*	*S. mitis*	2.0	Contig	No
SK575	AICU00000000	*S. mitis*	*S. mitis*	2.0	Contig	No
SK578	JPFY00000000	*S. mitis*	*S. mitis*	2.1	Contig	Yes
SK579	AJJL00000000	*S. mitis*	*S. mitis*	2.0	Contig	No
SK597	AEDV00000000	*S. mitis*	*S. mitis*	2.0	Contig	No
SK608	JPFZ00000000	*S. mitis*	*S. mitis*	2.1	Contig	Yes
SK616	AICR00000000	*S. mitis*	*S. mitis*	2.0	Contig	No
SK629	JPFU00000000	*S. mitis*	*S. mitis*	2.2	Contig	Yes
SK637	JPFX00000000	*S. mitis*	*S. mitis*	1.9	Contig	Yes
SK642	JPFW00000000	*S. mitis*	*S. mitis*	2.0	Contig	Yes
SK667	JPFV00000000	*S. mitis*	*S. mitis*	2.1	Contig	Yes
SK95	AFUB00000000	*S. mitis*	*S. oralis* subsp. *dentisani*	2.0	Contig	No
SPAR10	ALCH00000000	*S. mitis*	*S. infantis*	1.8	Contig	No
SVGS_061	CP014326	*S. mitis*	*S. mitis*	2.2	Complete	Yes
