# Supplementary material for: A Pangenome Approach for Discerning Species-Unique Gene Markers for Identifications of Streptococcus pneumoniae and Streptococcus pseudopneumoniae
Source: Front Cell Infect Microbiol. 2020 May 19;10:222. doi: 10.3389/fcimb.2020.00222 (PMC7248185; doi:10.3389/fcimb.2020.00222)
Supplement: Supplementary file 2 [file Table_2.pdf]

Supplementary Table S2. List of genome sequences of *S. pneumoniae* (n=42) and *S. pseudopneumoniae* (n=29) available in GenBank by November 22, 2019 that were not included in the pangenome analysis.

Strain	Accesion number	GenBank species designation	Cluster analysis ANIb	Size (Mb)	Level
335	CP026670	*S. pneumoniae*	*S. pneumoniae*	2.2	Complete
521	CP036529	*S. pneumoniae*	*S. pneumoniae*	2.0	Complete
947	LR129841	*S. pneumoniae*	*S. pneumoniae*	2.1	Complete
4496	LR129840	*S. pneumoniae*	*S. pneumoniae*	2.2	Complete
4559	LR595848	*S. pneumoniae*	*S. pneumoniae*	2.2	Complete
11A	CP018838	*S. pneumoniae*	*S. pneumoniae*	2.1	Complete
180-15	LR129844	*S. pneumoniae*	*S. pneumoniae*	2.0	Complete
180-2	LR129843	*S. pneumoniae*	*S. pneumoniae*	2.0	Complete
19F	CP025076	*S. pneumoniae*	*S. pneumoniae*	2.1	Complete
2245STDY5699475	LR536831	*S. pneumoniae*	*S. pneumoniae*	2.1	Complete
2245STDY6106635	LR536837	*S. pneumoniae*	*S. pneumoniae*	2.2	Complete
2245STDY6178787	LR216060	*S. pneumoniae*	*S. pneumoniae*	2.2	Complete
4041STDY6583227	LS483450	*S. pneumoniae*	*S. pneumoniae*	2.2	Complete
4041STDY6836166	LS483451	*S. pneumoniae*	*S. pneumoniae*	2.2	Complete
4041STDY6836167	LS483448	*S. pneumoniae*	*S. pneumoniae*	2.1	Complete
4041STDY6836169	LS483523	*S. pneumoniae*	*S. pneumoniae*	2.1	Complete
4041STDY6836170	LS483449	*S. pneumoniae*	*S. pneumoniae*	2.1	Complete
55896440-41bd-11e5-998e-3c4a9275d6c6	LR216065	*S. pneumoniae*	*S. pneumoniae*	2.1	Complete
569492b0-41bd-11e5-998e-3c4a9275d6c6	LR216064	*S. pneumoniae*	*S. pneumoniae*	2.1	Complete
ASP0581	AP019192	*S. pneumoniae*	*S. pneumoniae*	2.2	Complete
ATCC 49619	AP018938	*S. pneumoniae*	*S. pneumoniae*	2.1	Complete
AUSMDU00010538	CP045931	*S. pneumoniae*	*S. pneumoniae*	2.1	Complete
b04a6400-1f66-11e7-b93e-3c4a9275d6c8	LR536843	*S. pneumoniae*	*S. pneumoniae*	2.1	Complete
D39V	CP027540	*S. pneumoniae*	*S. pneumoniae*	2.0	Complete
EF3030	CP035897	*S. pneumoniae*	*S. pneumoniae*	2.1	Complete
GPS_US_PATH396-sc-2296505	LR216050	*S. pneumoniae*	*S. pneumoniae*	2.1	Complete
HKU1-14	CP019299	*S. pneumoniae*	*S. pneumoniae*	2.0	Complete
HU-OH	AP018937	*S. pneumoniae*	*S. pneumoniae*	2.1	Complete
KK0981	AP017971	*S. pneumoniae*	*S. pneumoniae*	2.1	Complete
M16808	CP031245	*S. pneumoniae*	*S. pneumoniae*	2.1	Complete
M23734	CP031247	*S. pneumoniae*	*S. pneumoniae*	2.1	Complete
M26365	CP031248	*S. pneumoniae*	*S. pneumoniae*	2.2	Complete
M26368	CP031246	*S. pneumoniae*	*S. pneumoniae*	2.1	Complete
MDRSPN001	AP018391	*S. pneumoniae*	*S. pneumoniae*	2.0	Complete
NCTC11902	LS483417	*S. pneumoniae*	*S. pneumoniae*	2.1	Complete
NCTC12977	LR134294	*S. pneumoniae*	*S. pneumoniae*	2.1	Complete
NCTC13276	LS483390	*S. pneumoniae*	*S. pneumoniae*	2.0	Complete
NCTC7465	LN831051	*S. pneumoniae*	*S. pneumoniae*	2.1	Complete
NU83127	AP018936	*S. pneumoniae*	*S. pneumoniae*	2.2	Complete
R6CIB17	CP038808	*S. pneumoniae*	*S. pneumoniae*	2.0	Complete
SPN XDR SMC1710-32	CP025838	*S. pneumoniae*	*S. pneumoniae*	2.1	Complete

Xen35	CP025256	*S. pneumoniae*	*S. pneumoniae*	2.2	Complete
5305	PDEO00000000	*S. pseudopneumoniae*	*S. pseudopneumoniae*	2.2	Contig
BHN868	SOQU00000000	*S. pseudopneumoniae*	*S. pseudopneumoniae*	2.2	Contig
BHN871	SOQT00000000	*S. pseudopneumoniae*	*S. pseudopneumoniae*	2.2	Contig
BHN877	SOQS00000000	*S. pseudopneumoniae*	*S. pseudopneumoniae*	2.3	Contig
BHN879	SOQV00000000	*S. pseudopneumoniae*	*S. pseudopneumoniae*	2.2	Contig
BHN880	SOQR00000000	*S. pseudopneumoniae*	*S. pseudopneumoniae*	2.3	Contig
BHN881	SOQQ00000000	*S. pseudopneumoniae*	*S. pseudopneumoniae*	2.2	Scaffold
BHN885	SOQP00000000	*S. pseudopneumoniae*	*S. pseudopneumoniae*	2.2	Scaffold
BHN886	SOQO00000000	*S. pseudopneumoniae*	*S. pseudopneumoniae*	2.2	Scaffold
BHN890	SOQN00000000	*S. pseudopneumoniae*	*S. pseudopneumoniae*	2.4	Contig
BHN891	SOQM00000000	*S. pseudopneumoniae*	*S. pseudopneumoniae*	2.2	Contig
BHN892	SOQL00000000	*S. pseudopneumoniae*	*S. pseudopneumoniae*	2.2	Scaffold
BHN893	SOQK00000000	*S. pseudopneumoniae*	*S. pseudopneumoniae*	2.2	Scaffold
BHN912	SOQJ00000000	*S. pseudopneumoniae*	*S. pseudopneumoniae*	2.2	Scaffold
BHN913	SOQI00000000	*S. pseudopneumoniae*	*S. pseudopneumoniae*	2.2	Contig
BHN914	SOQH00000000	*S. pseudopneumoniae*	*S. pseudopneumoniae*	2.2	Scaffold
BHN915	SOQG00000000	*S. pseudopneumoniae*	*S. pseudopneumoniae*	2.2	Scaffold
BHN916	SOQF00000000	*S. pseudopneumoniae*	*S. pseudopneumoniae*	2.3	Scaffold
BHN918	SOQE00000000	*S. pseudopneumoniae*	*S. pseudopneumoniae*	2.2	Contig
BHN919	SOQD00000000	*S. pseudopneumoniae*	*S. pseudopneumoniae*	2.2	Contig
BHN920	SOQC00000000	*S. pseudopneumoniae*	*S. pseudopneumoniae*	2.2	Contig
BHN922	SOQB00000000	*S. pseudopneumoniae*	*S. pseudopneumoniae*	2.2	Contig
CipR71	PDEN00000000	*S. pseudopneumoniae*	*S. pseudopneumoniae*	2.1	Contig
EL2652N1	PTTL00000000	*S. pseudopneumoniae*	*S. pseudopneumoniae*	2.2	Contig
NTPn 138	LTCN00000000	*S. pseudopneumoniae*	*S. pseudopneumoniae*	2.1	Scaffold
Spain2270	RAHZ00000000	*S. pseudopneumoniae*	*S. pseudopneumoniae*	2.2	Contig
Spain3473	PTTJ00000000	*S. pseudopneumoniae*	*S. pseudopneumoniae*	2.2	Contig
Spain939	PTQV00000000	*S. pseudopneumoniae*	*S. pseudopneumoniae*	2.4	Contig
Spain9880	PTTK00000000	*S. pseudopneumoniae*	*S. pseudopneumoniae*	2.2	Contig
