# Supplementary material for: A Pangenome Approach for Discerning Species-Unique Gene Markers for Identifications of Streptococcus pneumoniae and Streptococcus pseudopneumoniae
Source: Front Cell Infect Microbiol. 2020 May 19;10:222. doi: 10.3389/fcimb.2020.00222 (PMC7248185; doi:10.3389/fcimb.2020.00222)
Supplement: Supplementary file 3 [file Table_3.pdf]

Supplementary Table S3. Genomic information of genome sequences of *S. pseudopneumoniae* performed in the study.

Strain	ANIb (%)[a]	Accession Number	No. scaffolds (> 500 bp)	N50	Total length (bp)	Coverage	CDSs (total)	N's	GC (%)	Isolation source	Geographic origin
CCUG 47366	98.6	RXXW00000000	74	70, 251	2, 152, 464	784	2, 270	386	39.9	Missing	Sweden:Växjö
CCUG 50868	97.3	RXXV00000000	84	62, 624	2, 211, 663	821	2, 329	146	39.7	Human respiratory tract	Missing
CCUG 50871	98.8	RXXU00000000	77	53, 660	2, 112, 424	611	2, 212	20	39.9	Human respiratory tract	Missing
CCUG 61551	98.2	RXXT00000000	79	55, 725	2, 137, 952	607	2, 245	56	39.9	Human sputum	Sweden: Karlstad
CCUG 63793	97.5	RXXS00000000	84	53, 172	2, 248, 710	1, 196	2, 360	34	39.7	Human sputum	Sweden:Gothenburg
CCUG 64062	96.8	RXXR00000000	55	82, 262	2, 16,5 344	845	2, 281	245	39.9	Human sputum	Sweden:Kalmar
CCUG 70988	97.7	RXXQ00000000	95	48, 502	2, 175, 673	995	2, 290	67	39.9	Human sputum	Sweden:Gothenburg
CCUG 71653	98.5	RXXP00000000	83	56, 738	2, 195, 012	785	2, 312	200	39.8	Human sputum	Sweden:Gothenburg
CCUG 71996	98.5	RXXN00000000	83	54, 340	2, 169, 308	253	2, 298	92	39.8	Human sputum	Sweden:Trollhättan
CCUG 72018	97.2	RXXM00000000	78	47, 531	2, 128, 633	1, 020	2, 228	125	39.9	Human sputum	Sweden:Gothenburg
CCUG 72019	98.5	RXXL00000000	79	49, 224	2, 119, 718	828	2, 221	226	39.9	Human sputum	Sweden:Gothenburg
CCUG 72028	96.9	RXXK00000000	61	85, 433	2, 171, 385	960	2, 262	63	39.8	Human sputum	Sweden:Gothenburg
CCUG 72029	97.0	RXXJ00000000	70	90, 733	2, 189, 998	1, 063	2, 301	342	39.7	Human sputum	Sweden:Gothenburg
CCUG 72040	96.9	RXXI00000000	76	64, 040	2, 175, 171	956	2, 267	307	39.8	Human sputum	Sweden:Borås

[a]ANIb similarity values were calculated bidirectionally against the genome sequence of *S. pseudopneumoniae* CCUG 49455^T (NZ_ AICS00000000).
Whole genome sequences were determined.using an Illumina MiSeq platform (Eurofins Genomics, Konstanz, Germany)
