# Supplementary material for: A Pangenome Approach for Discerning Species-Unique Gene Markers for Identifications of Streptococcus pneumoniae and Streptococcus pseudopneumoniae
Source: Front Cell Infect Microbiol. 2020 May 19;10:222. doi: 10.3389/fcimb.2020.00222 (PMC7248185; doi:10.3389/fcimb.2020.00222)
Supplement: Supplementary file 4 [file Table_4.pdf]

Supplementary Table S4. Pangenome gene distribution between *S. pneumoniae* and *S. pseudopneumoniae*.

***S.pneumoniae – S. pseudopneumoniae* genomes (n=45)**		
	**Gene clusters (n)**	**Gene clusters (%)**
Core	887	16.7
Soft-core	1,302	24.5
Cloud	2,463	46.4
Shell	1,542	29.1
Pangenome	5,307	100
