# Supplementary material for: A Pangenome Approach for Discerning Species-Unique Gene Markers for Identifications of Streptococcus pneumoniae and Streptococcus pseudopneumoniae
Source: Front Cell Infect Microbiol. 2020 May 19;10:222. doi: 10.3389/fcimb.2020.00222 (PMC7248185; doi:10.3389/fcimb.2020.00222)
Supplement: Supplementary file 5 [file Table_5.pdf]

Supplementary Table S5. BLASTN analysis of the gene markers for *S. pneumoniae* and *S. pseudopneumoniae* against additional genome sequences available in GenBank by November 2019, as well as genome sequences of 20 *S. pneumoniae* and 14 *S. pseudopneumoniae* clinical strains that were not included in the pangenome analysis. The numbers indicate number of BLASTN hits of the gene markers against a set of genome sequences.

	S. pneumoniae		***S. pseudopneumoniae***	
	**NCBI genomes**	**Clinical strains[a]**	**NCBI genomes**	**Clinical strains[b]**
**Gene marker**	**n=42**	**n=20**	**n=29**	**n=14**
Pneumo_127	41	20	0	0
Pneumo_436	42	20	0	0
Pneumo_1011	40	20	2	0
Pneumo_1012	40	20	2	0
Pneumo_1013	40	20	2	0
Pneumo_1014	40	20	2	0
Pneumo_1361	42	20	0	0
Pneumo_1362	42	20	0	0
Pneumo_1961	41	20	1	0
Pneumo_1964	42	20	1	0
Pseudo_228	0	0	29	14
Pseudo_231	0	0	29	14
Pseudo_232	0	0	29	14
Pseudo_641	0	0	29	14
Pseudo_764	0	0	37	14
Pseudo_899	0	0	29	14
Pseudo_901	0	0	29	14
Pseudo_902	0	0	29	14
Pseudo_1933	0	0	26	14

[a]Salvà-Serra *et al*. 2017.
[b]This study.
