# Supplementary material for: A Pangenome Approach for Discerning Species-Unique Gene Markers for Identifications of Streptococcus pneumoniae and Streptococcus pseudopneumoniae
Source: Front Cell Infect Microbiol. 2020 May 19;10:222. doi: 10.3389/fcimb.2020.00222 (PMC7248185; doi:10.3389/fcimb.2020.00222)
Supplement: Supplementary file 6 [file Table_6.pdf]

Supplementary Table S6. List of *S. pneumoniae* and *S. pseudopneumoniae* unique gene markers and species-specific peptide matches detected by LC-MS/MS analysis.

**Species**	**Unique Gene**	**Peptide matches**
S. pneumoniae	Pneumo_127	No hit
	Pneumo_436	No hit
	Pneumo_1011	4
	Pneumo_1012	No hit
	Pneumo_1013	No hit
	Pneumo_1014	1
	Pneumo_1361	1
	Pneumo_1362	No hit
	Pneumo_1961	1
	Pneumo_1964	1
S. pseudopneumoniae	Pseudo_228	2
	Pseudo_231	No hit
	Pseudo_232	1
	Pseudo_641	No hit
	Pseudo_899	No hit
	Pseudo_901	No hit
	Pseudo_902	No hit
	Pseudo_1764	No hit
	Pseudo_1933	1
