# Supplementary material for: A Pangenome Approach for Discerning Species-Unique Gene Markers for Identifications of Streptococcus pneumoniae and Streptococcus pseudopneumoniae
Source: Front Cell Infect Microbiol. 2020 May 19;10:222. doi: 10.3389/fcimb.2020.00222 (PMC7248185; doi:10.3389/fcimb.2020.00222)
Supplement: Supplementary file 9 [file Data_Sheet_1.pdf]

Supplementary Figure S1. Phylogenetic tree of pair-wise genomic Average Nucleotide Identity (ANI) similarities based on the BLAST algorithm (ANIb) using the JspeciesWS between all genome sequences of 32 closed genomes of *S. pneumoniae*, 36 S. *pseudopneumoniae* and 65 *S. mitis*, as well as the type strain of*, S. infantis*, *S. oralis*, *S. oralis* subsp. *tigurinus* and *S. oralis* subsp. *dentisani.*

Supplementary Figure S2. Distribution of *S. pseudopneumoniae* gene markers on 29 clinical strains classified as *S. pseudopneumoniae.*

Supplementary information

Materials and methods

PCR-amplification

For amplification of the unique genes for both *S. pneumoniae* and *S. pseudopneumoniae*, the reaction mixture for PCR-assays included 0.1 to 10 ng of DNA template, 1X GoTaq Green Master mix (Promega Corporation, Madison, WI, USA), 1 µM concentration of each amplification-primer, in a total volume of 25 µL. The PCR conditions were as follows: initial denaturation at 95 °C for 5 min; followed by 30 cycles of denaturation at 95 °C for 30 sec, primer-annealing at 55 °C for 30 sec, and primer-extension at 72 °C for 90 sec; and a final elongation-period at 72°C for 5 min. PCR-products were resolved by electrophoresis in 1% agarose gels at 70 V for 20 min and stained afterwards with GelRed™ (Biotium, Hayward, CA, USA).

Optochin testing

Bacterial cell suspensions, with MacFarland 0.5 standardized turbidity, were prepared and inoculated onto Mueller-Hinton agar supplemented with 5% defibrinated horse blood (MH-F agar) (Substrate Division, Clinical Microbiological Laboratory, Sahlgrenska University Hospital, Gothenburg, Sweden). A disc of optochin (5 µg) (Oxoid) was added to each plate. Both plates were incubated overnight, one at 36 °C with 5% CO_2 and the other one at 37 °C in aerobic conditions. Sensitivity zones less than or equal to 14 mm were considered to indicate resistance to optochin. *S. pneumoniae* CCUG 28588^T and *S. pseudopneumoniae* CCUG 49455^T were used as controls.
